# Dual-barrier mechanism of superinfection exclusion in Newcastle disease virus: nucleoprotein and hemagglutinin-neuraminidase cooperate to block secondary infection

**DOI:** 10.1128/mbio.03545-25

**Published:** 2026-10-14

**Authors:** Kejia Lu, Xuhong Cao, Zhengwu Chang, Xi Wei, Sa Xiao, Zengqi Yang, Haijin Liu

**Affiliations:** 1 College of Veterinary Medicine, Northwest A&F University 12469, Yangling, Shaanxi, China; 2 Laboratory Animal Center, Xi'an Jiaotong University Health Science Center 105871, Xi'an, Shaanxi, China; 3 Engineering Research Center of Efficient New Vaccines for Animals, Ministry of Education, Yangling, China; 4 Key Laboratory of Ruminant Disease Prevention and Control (West), Ministry of Agriculture and Rural Affairs, Yangling, China; Tsinghua University, Beijing, China

**Keywords:** superinfection exclusion, Newcastle disease virus, nucleoprotein, hemagglutinin-neuraminidase, viral interference, antiviral strategies

## Abstract

Superinfection exclusion (SIE) is a well-documented phenomenon in virology, wherein a primary viral infection prevents subsequent infection by the same or closely related viruses through various mechanisms. This phenomenon has been observed with Newcastle disease virus (NDV), a member of the Paramyxoviridae family. However, the molecular mechanisms underlying NDV-mediated SIE remain unclear. In this study, we elucidate a dual-barrier mechanism of NDV-mediated SIE, involving both the nucleoprotein (NP) and the hemagglutinin-neuraminidase (HN) glycoprotein. Our findings demonstrate that NDV SIE is initiated within 2 h of primary infection and intensifies over time, independent of viral virulence and host cell type. The NP protein forms an early cytoplasmic barrier by sequestering incoming NP subunits, while HN-neuraminidase activity at the viral membrane cleaves sialic acid receptors, preventing secondary virion adsorption. Furthermore, our data reveal that NDV-mediated SIE is specific to intraspecies interactions and does not extend to heterologous viruses, such as vesicular stomatitis virus.

**IMPORTANCE:**

Superinfection exclusion (SIE) is a crucial viral strategy for limiting co-infection and ensuring the dominance of a single viral strain. The identification of the dual-barrier mechanism in Newcastle disease virus (NDV), involving both the nucleoprotein (NP) and hemagglutinin-neuraminidase (HN) proteins, significantly enhances our understanding of viral defense mechanisms. By elucidating how SIE operates at both the cytoplasmic and membrane levels, this study not only provides a mechanistic framework for NDV interference but also opens avenues for therapeutic applications. The discovery that NP and HN act synergistically to block secondary infection presents new strategies for combating viral co-infection in veterinary medicine and potentially in human viruses. Furthermore, understanding the viral determinants involved in SIE could guide the development of antiviral therapies that exploit these mechanisms to prevent superinfection and reduce viral spread.

## INTRODUCTION

Superinfection exclusion (SIE) is a widely observed phenomenon in virology, wherein a host cell infected by a primary virus becomes resistant to or completely blocks secondary infection by the same virus or closely related strains. This mechanism has been documented in a variety of plant and animal viruses, highlighting its significance across diverse viral families (1–8). Notably, SIE has been reported in several critical human and animal pathogens, including human immunodeficiency virus 1, hepatitis C virus, West Nile virus, herpes simplex virus 1, vesicular stomatitis virus (VSV), Chikungunya virus, influenza A virus, and pseudorabies virus (9–16). The blockade is typically initiated through receptor sequestration or rapid depletion of intracellular replication factors; if the secondary virus penetrates this first barrier, occupation of essential complexes and virus-induced remodeling of the cellular environment halt downstream events, such as entry, transcription, or integration, thereby ensuring that only the primary virus completes its replication cycle (1, 2, 4, 6, 9, 13, 15–20).

SIE has also found practical applications in both agricultural and medical fields. In agriculture, SIE is utilized for cross-protection, where prior infection with a mild strain of a virus prevents subsequent infection by more virulent strains, as demonstrated with Citrus tristeza virus and Pepino mosaic virus (21, 22). In medical virology, SIE is leveraged in therapeutic strategies, such as the use of defective interfering particles that compete for viral replication resources, showing promise as antiviral agents against influenza and other viral infections (23).

Newcastle disease virus (NDV) is a member of the genus Orthoavulavirus within the family Paramyxoviridae (24). The virulent strains pose a great risk for poultry. SIE has also been documented for Newcastle disease virus. As early as 1961, Durand (25) reported that viable NDV establishes strain-specific interference, and Bratt and Rubin (26, 27) later showed that this blockade is achieved by sequential receptor sequestration and occupation of intracellular replication sites. Using a recombinant La Sota strain expressing red fluorescent protein, Li et al. (28) visualized the process at single-cell resolution and demonstrated that it is independent of virulence and provides a standardized platform for mechanistic studies of NDV SIE (25–28). However, the mechanism of NDV SIE remains unclear.

NDV genome consists of a single, non-segmented, negative-sense RNA that encodes six structural proteins: nucleoprotein (NP), phosphoprotein (P), matrix (M) protein, fusion (F) protein, hemagglutinin-neuraminidase (HN), and large (L) protein (29). The P gene also expresses two nonstructural proteins, V and W, through RNA editing (30). NP is the first and most abundant protein translated from the viral RNA. The protein is organized into two functional regions: a highly conserved N-terminal core (Ncore) that assembles into an ordered ribonucleoprotein (RNP) shell and a flexible C-terminal tail (Ntail) that protrudes from this shell and is exposed on the RNP surface (31, 32). Ncore provides all the determinants required for NP self-assembly and for wrapping genomic RNA, while Ntail constitutes the binding platform that recruits the P protein to the nascent RNP (33, 34). Together with the L polymerase, NP and P cooperatively encapsidate viral RNA to form the minimal replication unit of the virus. The envelope-associated HN glycoprotein performs two sequential tasks that are essential for the NDV life cycle. Its hemagglutinin (HA) activity mediates high-affinity attachment to sialic acid (SA) moieties on the host cell, and its neuraminidase (NA) activity subsequently removes SA from the budding site and progeny virions, preventing self-aggregation and ensuring efficient release of infectious particles (35). Alanine substitution at either leucine 160 or phenylalanine 220 reduces hemadsorption (HAd) activity to approximately 20% and completely abolishes fusion promotion (36), while replacement of proline 93 with leucine almost entirely eliminates neuraminidase activity (37, 38). Residue 198 is particularly critical because it simultaneously governs receptor recognition, fusion capacity, and neuraminidase function (39–41).

In this study, we demonstrate a dual-barrier mechanism of NDV SIE. Using different fluorescently tagged recombinant viruses, we found that homologous exclusion begins within 2 h, strengthens with time, and is independent of virulence or cell type. In an early cytoplasmic phase, self-aggregating NP sequesters incoming NP subunits, thereby aborting replication complex formation, followed by a membrane phase where HN NA cleaves SA receptors to prevent adsorption of secondary virions. This dual-barrier mechanism provides new insights into the events of NDV SIE, highlighting potential therapeutic targets to disrupt the virus-host interaction and limit viral spread.

## RESULTS

### Superinfection exclusion of NDV

**Fig 1:**
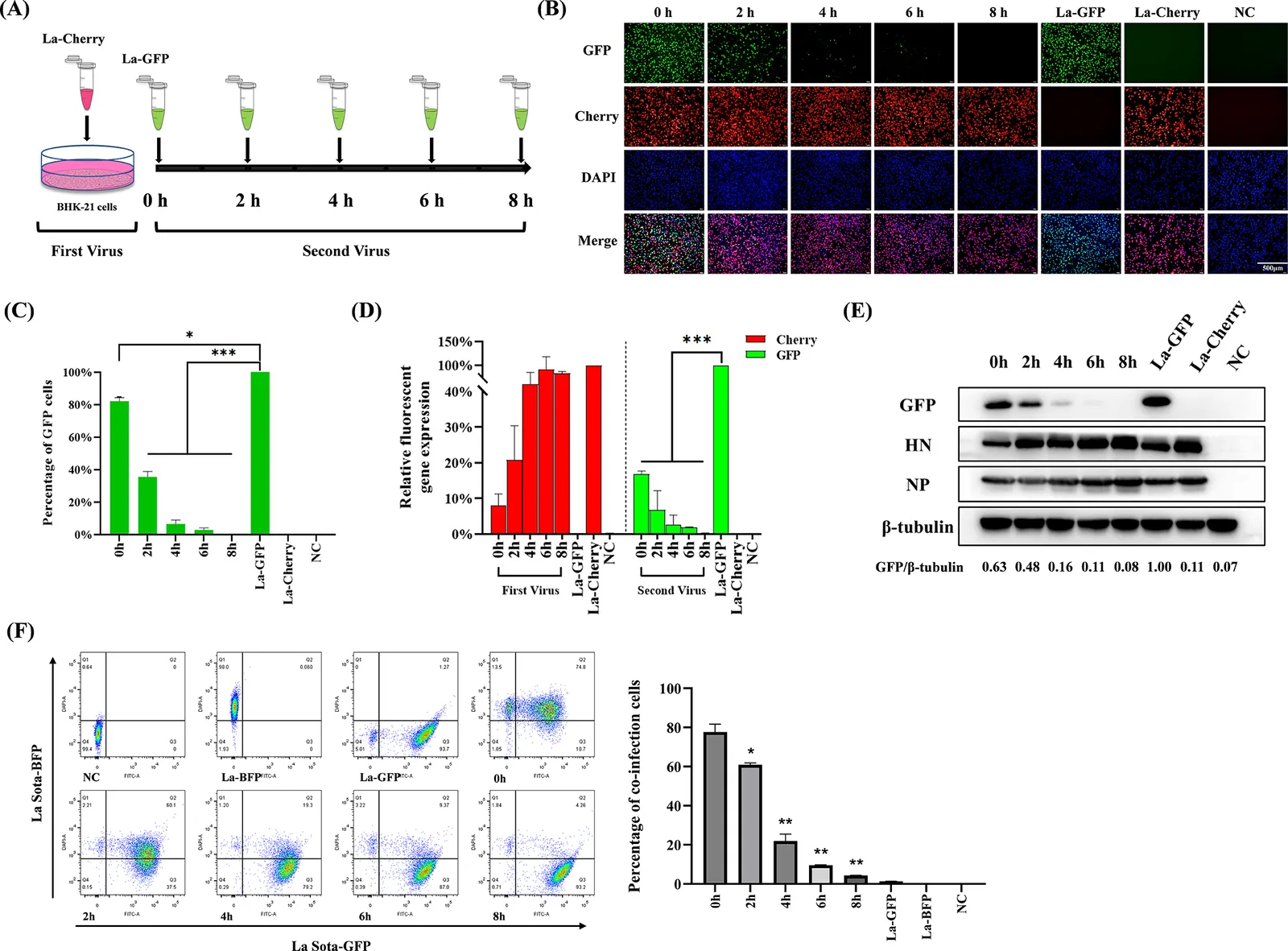
La Sota-Cherry excludes La Sota-GFP infection in BHK-21 cells. (**A**) Schematic representation of the virus infection. BHK-21 cells were infected with La Sota-Cherry (MOI = 5) and challenged with La Sota-GFP (MOI = 1) at 0-, 2-, 4-, 6-, or 8-h intervals. Controls consisted of single infections with La Sota-Cherry (MOI = 5) and La Sota-GFP (MOI = 1). NC, cells treated with the same conditions but not infected with the virus. Imaging and sampling were performed 24 h after the second or single virus infection. (**B**) Representative micrographs show fluorescence signal. The cells were fixed, and nuclei were stained with Hoechst 33342. Scale bar, 500 µm. (**C**) Percentage of GFP-positive cells counted from five random fields (*n* = 3). (**D**) The RNA of the fluorescent reporter gene was detected by qRT-PCR. As a loading control, 28s was used (*n* = 3). (**E**) Western blot analysis was performed on BHK-21 cells to assess viral protein expression. GFP protein levels relative to β-tubulin were quantified by densitometry. Data were normalized to La-GFP, which was set to 1. (**F**) BHK-21 cells were infected with La Sota-GFP (MOI = 5) and challenged with La Sota-BFP (MOI = 1) at 0-, 2-, 4-, 6-, or 8-h intervals; controls consisted of single infections with La Sota-GFP (MOI = 5) or La Sota-BFP (MOI = 1). NC, cells treated with the same conditions but not infected with the virus. Flow cytometry analysis of BFP and GFP expression. The co-infection rates were calculated as the percentage of cells co-expressing GFP and BFP, divided by the total number of GFP-positive cells for each group (*n* = 3). Data are given as means ± standard deviation from three independent experiments. *P* values were calculated using Student’s *t*-test. An asterisk indicates a comparison with the indicated control. *P < 0.05; **P < 0.01; ***P < 0.001; and ns, not significant.

**Fig 2:**
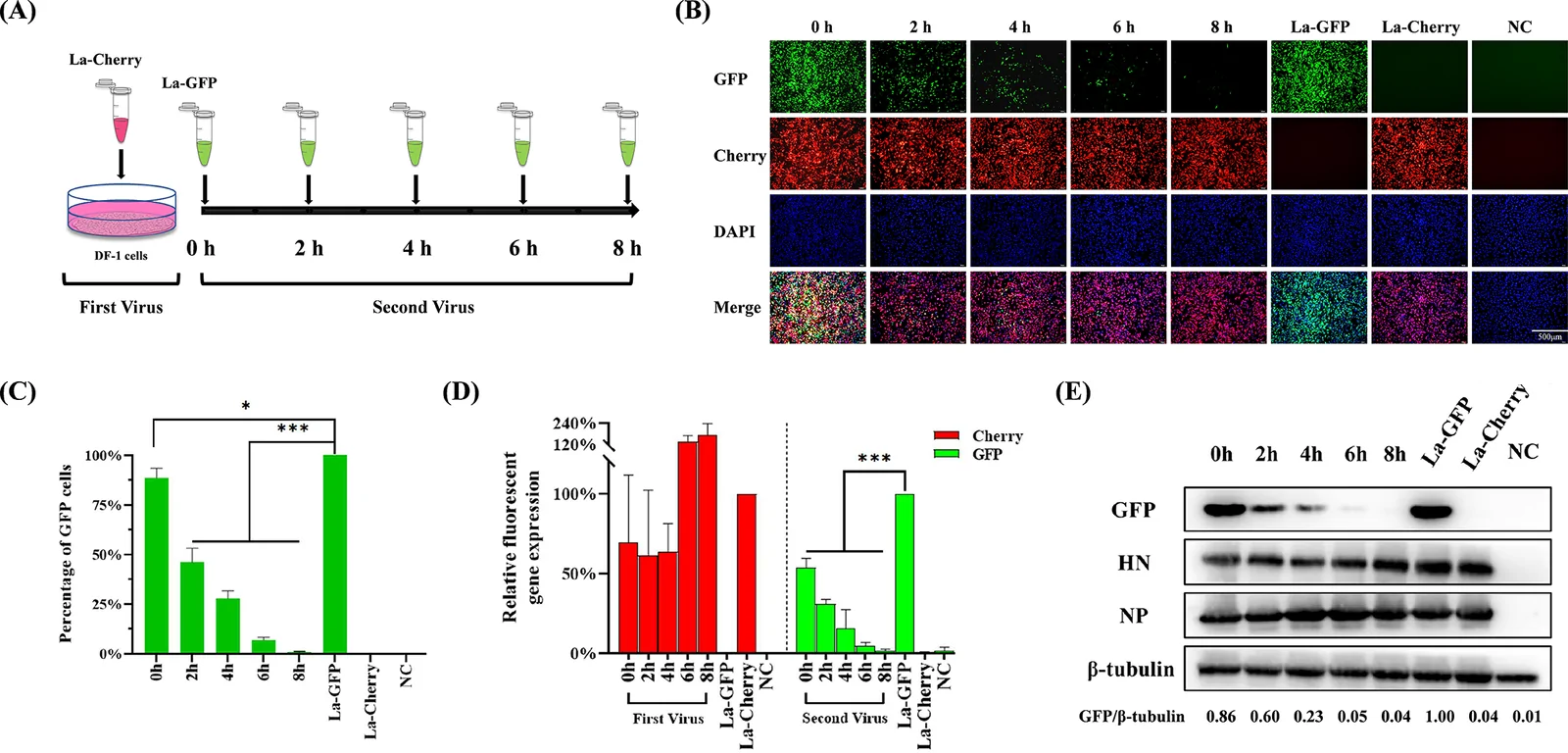
La Sota-Cherry excludes La Sota-GFP infection in DF-1 cells. (**A**) Schematic representation of the virus infection. DF-1 cells were infected with La Sota-Cherry (MOI = 5) and challenged with La Sota-GFP (MOI = 1) at 0-, 2-, 4-, 6-, or 8-h intervals. Controls consisted of single infections with La Sota-Cherry (MOI = 5) or La Sota-GFP (MOI = 1). NC, cells treated with the same conditions but not infected with the virus. Imaging and sampling were performed 24 h after the second or single virus infection. (**B**) Representative micrographs show fluorescence signal. The cells were fixed, and nuclei were stained with Hoechst 33342. Scale bar, 500 µm. (**C**) Percentage of GFP-positive cells counted from five random fields (*n* = 3). (**D**) The RNA of the fluorescent reporter gene was detected by qRT-PCR. As a loading control, 28s was used (*n* = 3). (**E**) Western blot analysis was performed on DF-1 cells to assess viral protein expression. GFP protein levels relative to β-tubulin were quantified by densitometry. Data were normalized to La-GFP, which was set to 1. Data are given as means ± standard deviation from three independent experiments. *P* values were calculated using Student’s *t*-test. An asterisk indicates a comparison with the indicated control. *P < 0.05; **P < 0.01; ***P < 0.001; and ns, not significant.

Superinfection exclusion refers to the phenomenon where the cell infected by a primary virus blocks subsequent infections by a second virus. To investigate whether NDV can induce SIE and explore the underlying mechanisms, NDV strains should be marked distinctively to facilitate the identification of cells infected by each individual virus. Using the NDV reverse genetics system, the recombinant La Sota strains carrying either Cherry (La Sota-Cherry), GFP (La Sota-GFP), or BFP (La Sota-BFP) fluorescent markers were rescued. Then, these strains were employed to establish a visual model to study NDV SIE. Initial experiments in NDV-susceptible BHK-21 cells showed that primary infection with La Sota-Cherry followed by secondary La Sota-GFP challenge at 0, 2, 4, 6, or 8 h post-infection (hpi) revealed suppression of secondary infection as early as 2 hpi of the first virus (Fig. 1A and B). Time-dependent attenuation of GFP fluorescence was observed, culminating in the disappearance of secondary infection signals below detection thresholds by 8 hpi (Fig. 1B). Quantitative analysis confirmed the progressive decline in La Sota-GFP-positive cells with increasing infection intervals (Fig. 1C). Molecular characterization at 24 h post-secondary infection further demonstrated progressively decreasing GFP RNA levels by RT-qPCR and corresponding reductions in GFP protein expression by western blotting (WB), indicating establishment of homologous virus exclusion within 2 h of primary infection (Fig. 1D and E). For precise quantification, flow cytometry analysis was performed using La Sota-GFP and La Sota-BFP. This analysis revealed that BFP-positive cells decreased from 88% (coinfection at 0 h) to 6% (8 h interval infection), and double-positive cells also declined from 74.8% to 4% over the same period (Fig. 1F). Using La Sota-GFP for primary infection followed by La Sota-Cherry challenge yielded consistent results, independent of the fluorescent protein expressed by the primary infecting virus (Fig. S1). When extended to DF-1 cells, identical experimental paradigms produced equivalent outcomes across multiple detection methods (Fig. 2; Fig. S2), confirming the cell-type independence of NDV-mediated SIE. Collectively, our data established that NDV initiates SIE in cells within 2 h of primary infection, exhibiting time-dependent intensification and cell-type independence.

### NDV mediates intraspecies but not cross-species SIE

Based on documented SIE between attenuated NDV La Sota strains, we next investigated whether an attenuated NDV strain could interfere with the infection by a virulent NDV strain. When BHK-21 cells were sequentially infected with La Sota-Cherry (attenuated strain) followed by the virulent C22-GFP strain at varying intervals (0–8 h), we observed progressively stronger inhibition of the virulent strain as the interval increased, with near-complete suppression at 8 h after the first infection (Fig. S3A through C). This phenomenon was further confirmed at both the transcriptional and translational levels by RT-qPCR and WB analyses, demonstrating the NDV SIE is independent of viral virulence (Fig. S3D and E).

**Fig 3:**
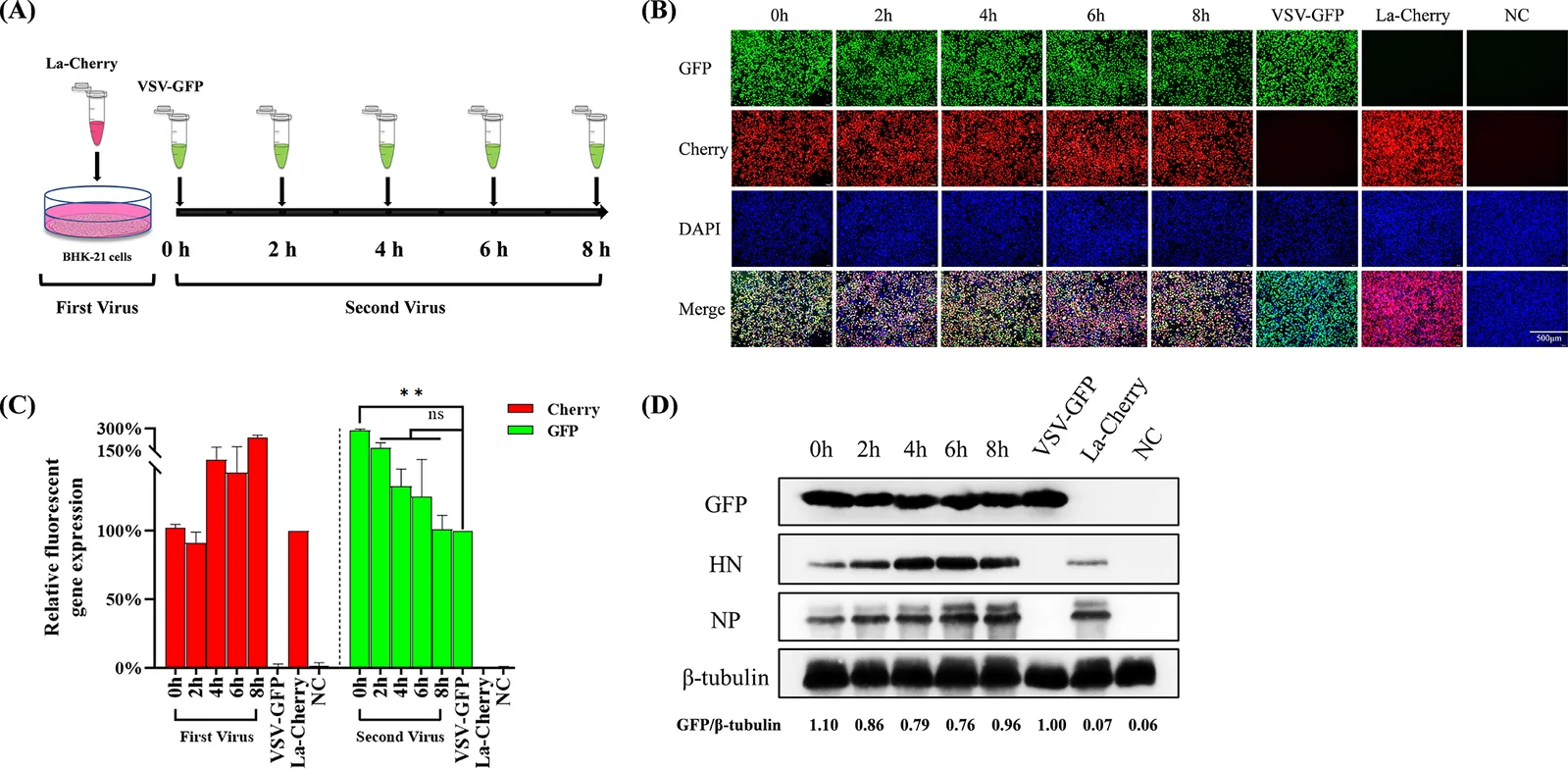
SIE is not observed between NDV and VSV. (**A**) Schematic representation of the virus infection. BHK-21 cells were infected with La Sota-Cherry (MOI = 5) and challenged with VSV-GFP (MOI = 1) at 0-, 2-, 4-, 6-, or 8-h intervals. Controls consisted of single infections with La Sota-Cherry (MOI = 5) or VSV-GFP (MOI = 1). NC, cells treated with the same conditions but not infected with the virus. Imaging and sampling were performed 24 h after the second or single virus infection. (**B**) Representative micrographs show fluorescence signal. The cells were fixed, and nuclei were stained with Hoechst 33342. Scale bar, 500 µm. (**C**) The RNA of the fluorescent reporter gene was detected by qRT-PCR. As a loading control, 28s was used (*n* = 3). (**D**) Western blot analysis was performed on BHK-21 cells to assess viral protein expression. HN and NP proteins indicated NDV infection. GFP protein levels relative to β-tubulin were quantified by densitometry. Data were normalized to VSV-GFP, which was set to 1. Data are given as means ± standard deviation from three independent experiments. *P* values were calculated using Student’s *t*-test. An asterisk indicates a comparison with the indicated control. *P < 0.05; **P < 0.01; ***P < 0.001; and ns, not significant.

Next, we investigated whether NDV-mediated SIE is restricted to NDV strains or extends to heterologous viruses. Using VSV-GFP, belonging to *Rhabdoviridae*, as a heterologous virus, we found no reduction in VSV-GFP infection efficiency even after an 8-h NDV pre-infection interval (Fig. 3A and B). Consistent results were obtained through viral RNA quantification and protein expression analysis, collectively demonstrating the absence of cross-species SIE between NDV and VSV (Fig. 3C and D).

Together, these results indicate that NDV-mediated SIE is virus-specific, effectively restricting both lentogenic and velogenic NDV strains, while showing no inhibitory effect on the heterologous virus VSV.

### NDV HN protein mediates SIE

These observations suggested that SIE likely involves viral self-factor expression during primary infection. Morrison and McGinnes (42) established in 1989 that expression of the NDV HN protein in cells confers resistance against both NDV and influenza virus challenge, positioning HN as a plausible mediator of NDV SIE.

**Fig 4:**
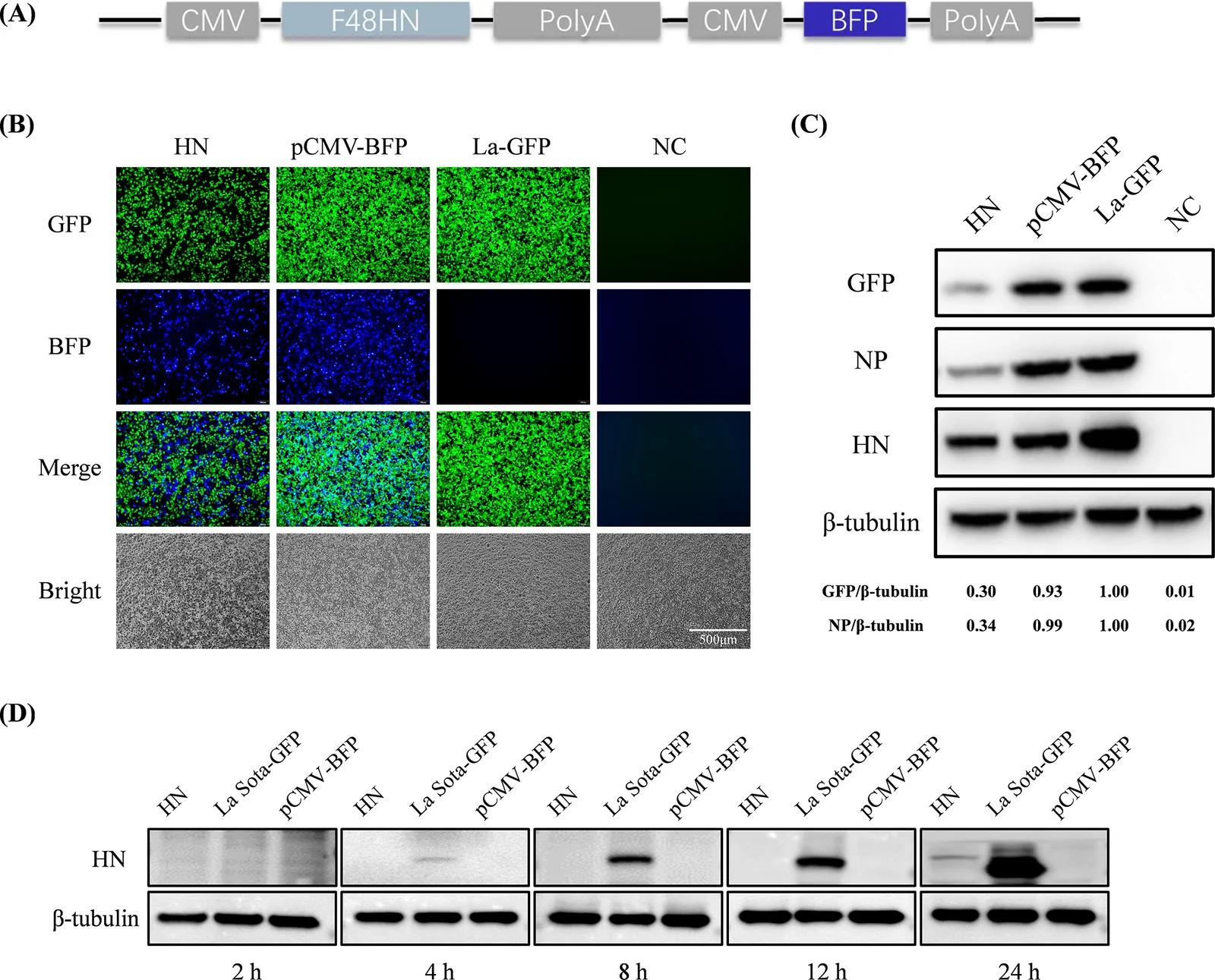
Pre-expression of HN blocks NDV infection. (**A**) Schematic representation of NDV HN plasmid. (**B**) BHK-21 cells were transfected with 1 µg of plasmids, pCMV-HN-BFP (HN) or pCMV-BFP. After 24 h, cells were infected with La Sota-GFP (MOI = 1) and analyzed at 24 hpi. NC, cells treated under the same conditions but without plasmid transfection and viral infection. Representative micrographs show fluorescence signal. Scale bar, 500 µm. (**C**) Western blot analysis was performed to assess GFP and viral protein expression. GFP and NP protein levels relative to β-tubulin were quantified by densitometry. Data were normalized to La-GFP, which was set to 1. (**D**) Comparison of HN expression following plasmid transfection and viral infection. BHK-21 cells were transfected with 1 μg of pCMV-HN-BFP (HN) or infected with La Sota-GFP (MOI = 5). At 2, 4, 8, 12, and 24 h post-treatment, cells were harvested for analysis. Cells transfected with 1 μg of pCMV-BFP served as the vector control. Western blot analysis was performed to assess HN protein expression. Data are given as means ± standard deviation from three independent experiments. *P* values were calculated using Student’s *t*-test. An asterisk indicates a comparison with the indicated control. *P < 0.05; **P < 0.01; ***P < 0.001; and ns, not significant.

To functionally characterize the role of HN in SIE, we engineered a plasmid containing BFP and HN genes, allowing us to track cells expressing HN via blue fluorescence (Fig. 4A). Transfected cells were challenged with La Sota-GFP. At 24 hpi, HN-overexpressing cells exhibited significantly reduced intensity of GFP signal (Fig. 4B). Corroborating this, WB analysis demonstrated markedly diminished viral GFP and NP protein expression in HN-expressing cells relative to controls, collectively establishing that HN pre-expression resisted NDV infection (Fig. 4C). In addition, the quantity of HN protein in the transfection group was substantially lower than that in NDV-infected cells (Fig. 4D). These findings suggest that the level of HN protein expression from NDV infection cell is sufficient to mediate SIE.

If the HN protein is the factor regulating NDV SIE, heterologous viruses that did not show SIE with NDV but express this protein may also exhibit SIE against NDV. Based on the absence of SIE between NDV and Peste des petits ruminants virus (PPRV), we constructed a chimeric PPRV expressing GFP and NDV HN protein. The HN gene of the NDV F48E9 strain was inserted between the leader and N gene within the PPRV Nigeria 75/1 vaccine strain backbone (Fig. S4A). This strain was rescued and named PPRV-HN-GFP. WB and immunofluorescence assay (IFA) confirmed robust NDV HN expression in PPRV-HN-GFP-infected cells, undetected in PPRV-GFP controls (Fig. S4B and C). Functional characterization verified that the HN protein, expressed by the PPRV, possessed membrane fusion-promoting function, hemagglutination, and neuraminidase activity, which were absent in PPRV-GFP (Fig. S4D through F).

**Fig 5:**
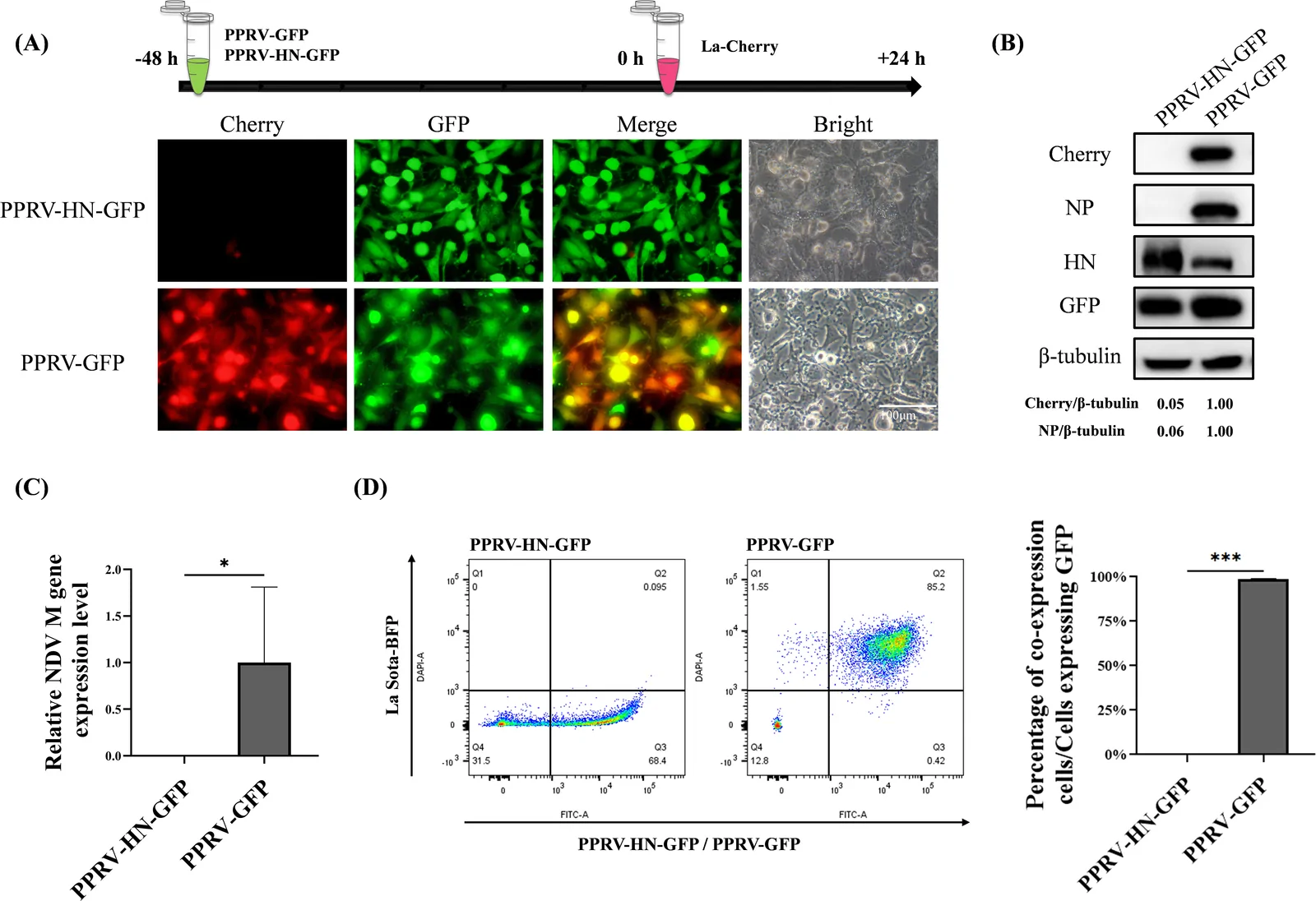
NDV HN protein expressed by recombinant PPRV confers potent SIE against NDV at the viral level. (**A**) BHK-21 cells were infected with either PPRV-GFP or PPRV-HN-GFP (MOI = 5) and challenged 48 h later with La Sota-Cherry (MOI = 1); imaging and sampling were performed 24 h after the second infection. Representative micrographs show fluorescence signal. Scale bar, 100 µm. (**B**) Western blot analysis was performed on BHK-21 cells 24 h after the second infection to assess viral protein expression. Cherry and NP protein levels relative to β-tubulin were quantified by densitometry. Data were normalized to PPRV-GFP, which was set to 1. (**C**) The relative RNA level of the NDV M gene was determined by qRT-PCR, normalized to the PPRV-GFP group. As an internal control, 28s was used (*n* = 3). (**D**) BHK-21 cells were infected with either PPRV-GFP or PPRV-HN-GFP (MOI = 5) and challenged 48 h later with La Sota-BFP (MOI = 1). Flow cytometry analysis of BFP and GFP expression. The co-infection rates were calculated as the percentage of cells co-expressing GFP and BFP, divided by the total number of GFP-positive cells for each group. Data are given as means ± standard deviation from three independent experiments. *P* values were calculated using Student’s *t*-test. An asterisk indicates a comparison with the indicated control. *P < 0.05; **P < 0.01; ***P < 0.001; and ns, not significant.

To verify the effect of HN-mediated SIE at the viral level, we utilized PPRV-HN-GFP or PPRV-GFP for primary infection, followed by La Sota-Cherry challenge. Because PPRV replicates more slowly than NDV in BHK-21 cells, we allowed PPRV infection to proceed for 48 h to ensure sufficient expression of the NDV HN protein encoded by PPRV-HN-GFP. Fluorescence microscopy revealed markedly reduced red fluorescence from La Sota-Cherry in cells pre-infected with PPRV-HN-GFP compared with those pre-infected with PPRV-GFP (Fig. 5A). WB analysis showed that expression of NDV Cherry and NP proteins was nearly undetectable in PPRV-HN-GFP-infected cells (Fig. 5B). Consistent with these findings, the M gene RNA of NDV was not detected, and flow cytometry quantified around 88% exclusion efficiency (Fig. 5C and D). Notably, the exclusion phenotype observed in the PPRV-HN-GFP system was more pronounced than that seen in the plasmid-transfection assay, consistent with the idea that HN expression in the context of viral infection may strengthen SIE. Together, these data demonstrated that HN protein mediates SIE of NDV.

### HN neuraminidase activity mediates NDV SIE

The HN protein may modulate SIE in two ways: by binding sialic acid through its HAd activity or by cleaving sialic acid through its NA activity, thereby interfering with the NDV sialic acid receptor-dependent entry process.

**Fig 6:**
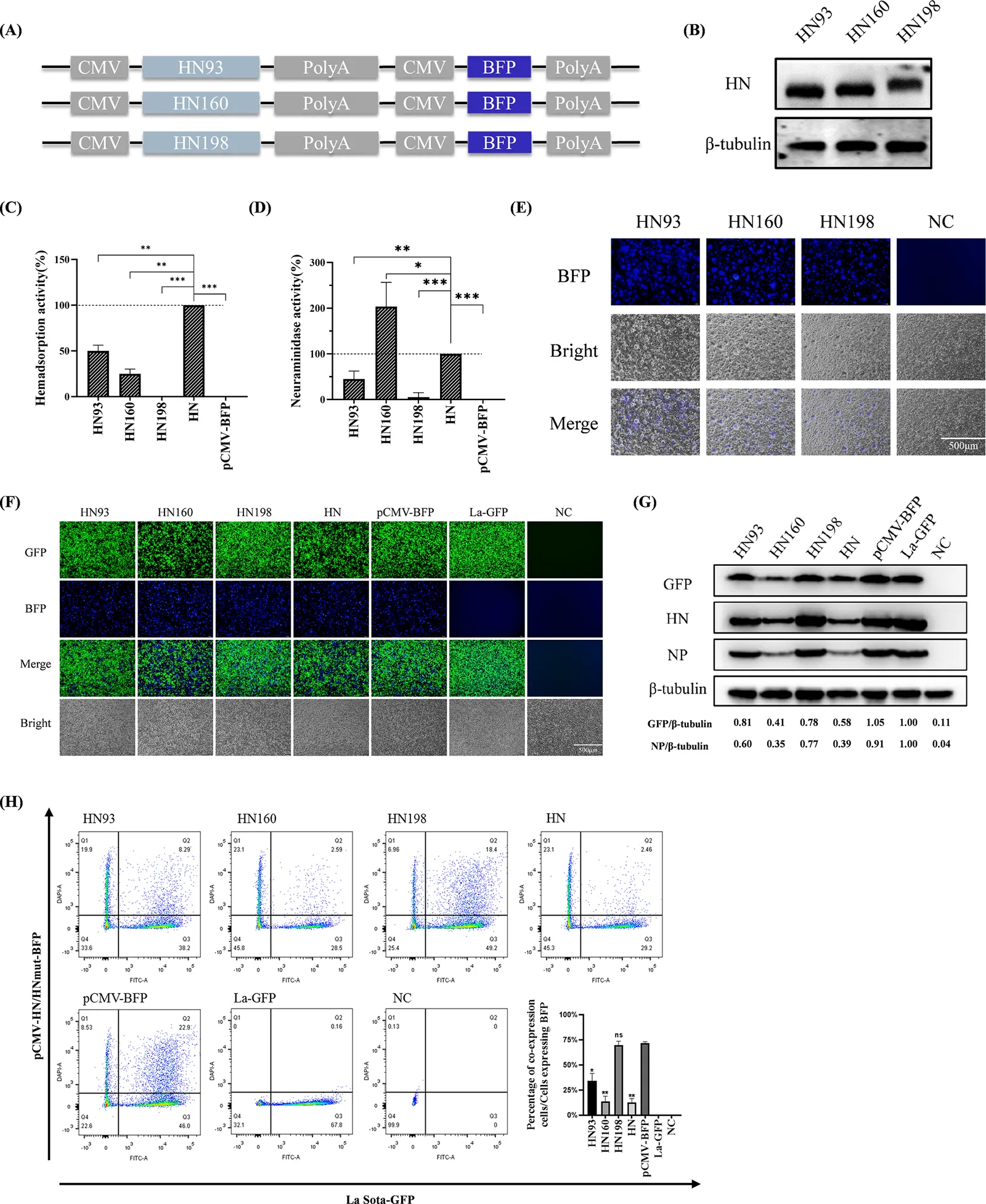
Interference of HN mutant proteins against NDV infection. (**A**) Schematic representation of NDV HN mutant plasmids. BHK-21 cells were transfected with HN mutants; 24 h post-transfection, protein was detected by Western blot (**B**), and HAd (**C**) and NA (**D**) activities were measured, respectively (*n* = 3). Data are normalized to wild-type HN activity (set at 100%). (**E**) BHK-21 cells were co-transfected with F and WT or mutant HN plasmids. At 24 h post-transfection, syncytia were examined. NC, cells co-transfected with F and the empty pCAGGS vector under the same conditions. (**F**) BHK-21 cells were transfected with 1 µg of plasmids encoding each HN mutant. After 24 h, cells were infected with La Sota-GFP (MOI = 1) and analyzed at 24 hpi. Representative micrographs show fluorescence signal. Scale bar, 500 µm. NC, cells treated under the same conditions but without plasmid transfection and viral infection. (**G**) Western blot analysis was performed to assess viral protein expression. GFP and NP protein levels relative to β-tubulin were quantified by densitometry. Data were normalized to La-GFP, which was set to 1. (**H**) Flow cytometry analysis of BFP and GFP expression. The infection rates of cells pre-expressing HN protein were calculated as the percentage of cells co-expressing GFP and BFP, divided by the total number of BFP-positive cells for each group. Data are given as means ± standard deviation from three independent experiments. *P* values were calculated using Student’s *t*-test. An asterisk indicates a comparison with the indicated control. *P < 0.05; **P < 0.01; ***P < 0.001; and ns, not significant.

Previous studies have shown that specific mutations in HN differentially affect these two activities: P93L reduces both HAd and NA activities, L160A decreases HAd activity while enhancing NA activity, and D198Q almost completely abolishes NA activity (36–41). Accordingly, to further investigate the role of HN in NDV SIE, we generated three corresponding HN mutants, designated HN93 (P93L), HN160 (L160A), and HN198 (D198Q). We then constructed plasmids expressing these HN mutants and confirmed expression of the corresponding proteins by western blotting (Fig. 6A and B). Subsequent functional analysis showed that, compared with wild-type (WT) HN, HN93 exhibited concomitant reductions in both HAd and NA activities, HN160 displayed reduced HAd activity but significantly enhanced NA activity, and HN198 was severely impaired in both activities, consistent with previous studies (Fig. 6C and D). Finally, when co-expressed with the F protein, these mutant HN proteins retained the ability to induce syncytium formation, indicating that they maintained fusion-promoting activity (Fig. 6E).

We next evaluated the impact of these mutants on NDV infection. Plasmids encoding the mutants were transfected into BHK-21 cells, followed by infection with La Sota-GFP 24 h later. Fluorescence microscopy, western blotting, and flow cytometric analyses consistently showed that expression of either HN160 or WT HN markedly suppressed NDV infection, as evidenced by fewer GFP-positive cells, reduced GFP and NP protein levels, and co-infection rates of approximately 10% (Fig. 6F through H). HN93 retained only weak inhibitory activity, with a co-infection rate of approximately 40%, whereas HN198 showed no significant suppressive effect on NDV infection (Fig. 6F through H). These results indicated that NA activity may relate to HN regulating NDV SIE.

**Fig 7:**
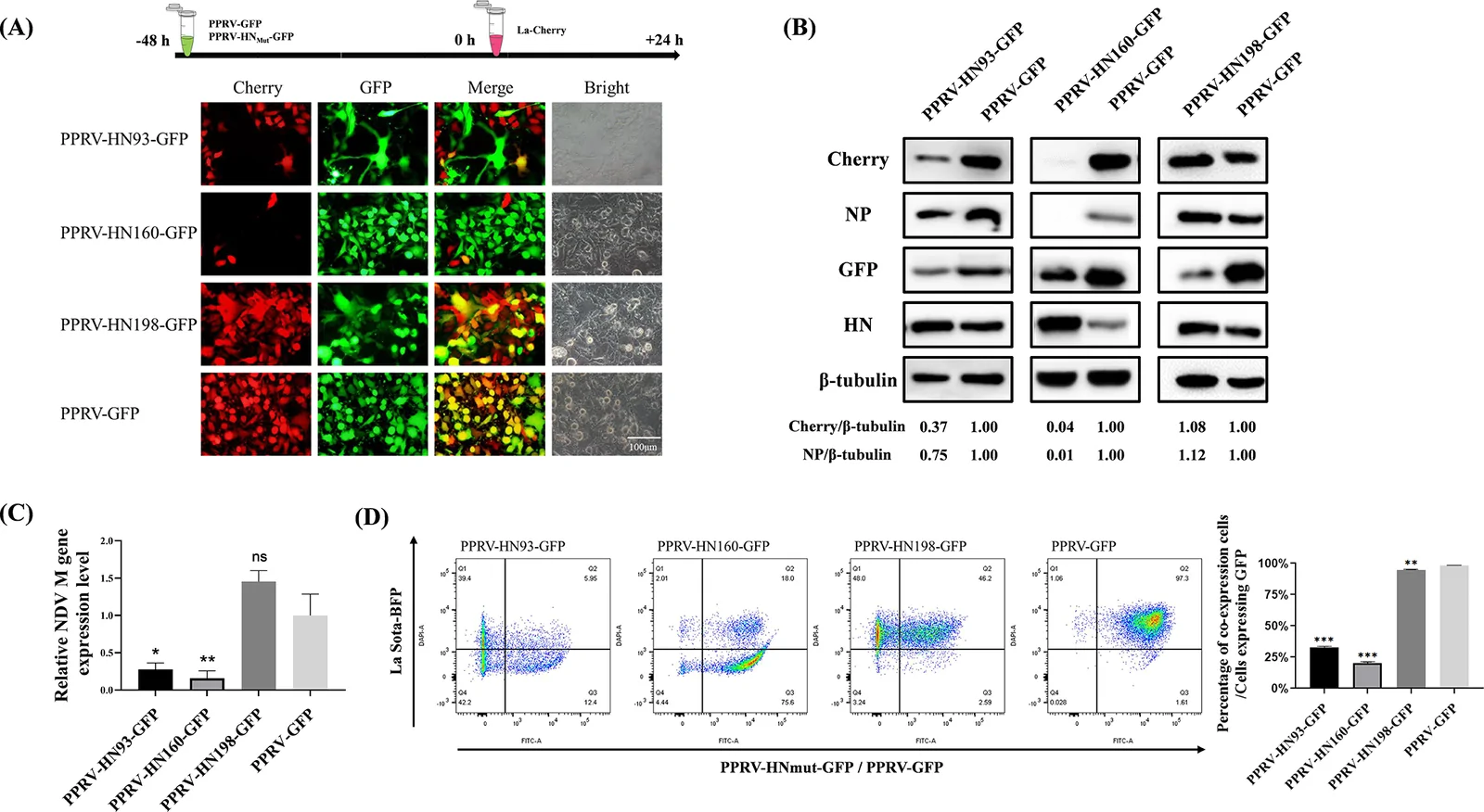
HN NA activity dictates NDV SIE. (**A**) BHK-21 cells were infected with PPRV-GFP and PPRV-GFP expressing NDV HN mutants (MOI = 5), respectively, and challenged 48 h later with La Sota-Cherry (MOI = 1); imaging and sampling were performed 24 h after the second infection. Representative micrographs show fluorescence signal. Scale bar, 100 µm. (**B**) Western blot analysis was performed on BHK-21 cells 24 h after the second infection to assess viral protein expression. Cherry and NP protein levels relative to β-tubulin were quantified by densitometry. Data were normalized to PPRV-GFP, which was set to 1. (**C**) The relative RNA level of the NDV M gene was determined by qRT-PCR, normalized to the PPRV-GFP group. As an internal control, 28s was used (*n* = 3). (**D**) BHK-21 cells were infected with either PPRV-GFP or PPRV-HNmut-GFP (MOI = 5) and, 48 h later, challenged with La Sota-BFP (MOI = 1). Flow cytometry analysis of BFP and GFP expression. The co-infection rates were calculated as the percentage of cells co-expressing GFP and BFP, divided by the total number of GFP-positive cells for each group. Data are given as means ± standard deviation from three independent experiments. *P* values were calculated using Student’s *t*-test. An asterisk indicates a comparison with the indicated control. *P < 0.05; **P < 0.01; ***P < 0.001; and ns, not significant.

To definitively validate this hypothesis, we generated recombinant virus PPRV-HN93-GFP, PPRV-HN160-GFP, and PPRV-HN198-GFP. After verification, these recombinant viruses were confirmed to express HN and its corresponding mutants (Fig. S5A). Relative to PPRV-HN-GFP (set as 100%), PPRV-HN93-GFP retained 56.3% HAd and 41% NA activity, PPRV-HN160-GFP exhibited reduced HAd (43%) but potentiated NA function (165.9%), while PPRV-HN198-GFP was deficient in both activities (Fig. S5B and C). Then, we assessed SIE between these recombinant viruses and NDV. Cells pre-infected with each recombinant virus were reinfected with NDV La Sota-Cherry or La Sota-BFP. At 24 hpi, analysis revealed that cells pre-infected with either PPRV-HN93-GFP or PPRV-HN160-GFP exhibited exclusion of NDV superinfection. Conversely, pre-infection with PPRV-HN198-GFP conferred no detectable SIE effect at the cellular, protein, or RNA level (Fig. 7). Furthermore, compared to the HN93 mutation, PPRV-expressing HN160 showed a more pronounced exclusion of NDV infection (Fig. 7). Given that HN160 exhibited weaker HAd activity but stronger NA activity than HN93, these results suggest that the HN protein regulates NDV SIE primarily through its neuraminidase function.

### NDV NP protein mediates SIE

**Fig 8:**
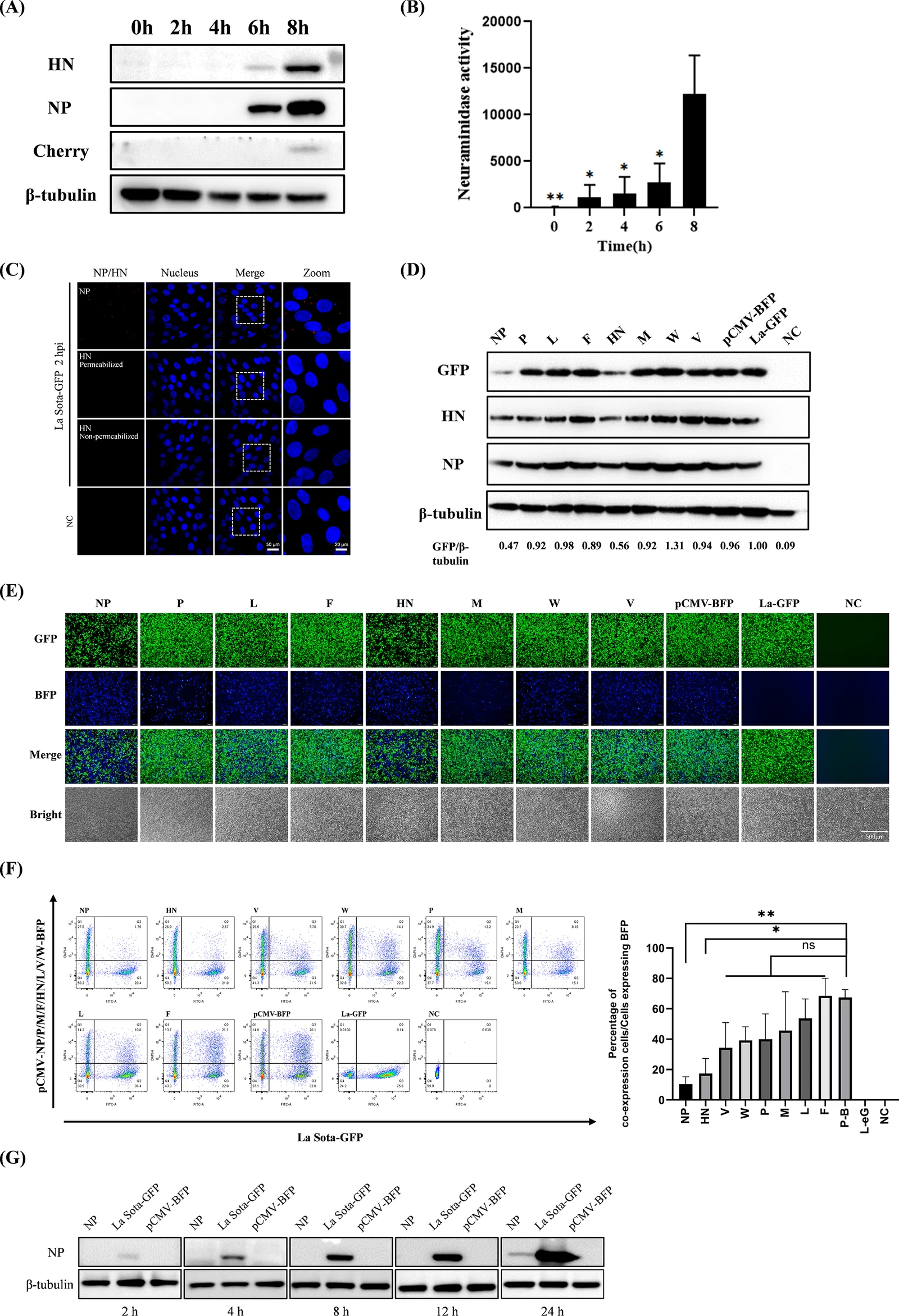
Like HN, NP protein mediates NDV SIE. (**A**) BHK-21 cells were infected with La Sota-Cherry (MOI = 5) and sampled at 0, 2, 4, 6, and 8 hpi. Expression of NDV NP and HN in La Sota-Cherry-infected cells was detected. (**B**) NA activity was determined (*n* = 3). (**C**) BHK-21 cells were infected with La Sota-GFP (MOI = 5) and fixed at 2 hpi. NDV NP and HN proteins were detected by immunofluorescence assay; HN was detected under both permeabilized and non-permeabilized conditions. Cell nuclei were counterstained with Hoechst 33342. NC, uninfected cells, subjected to the same experimental procedures, were included as a negative control. Scale bars: main image, 50 μm; inset, 20 μm. (**D**) BHK-21 cells were transfected with 1 µg of plasmids encoding each NDV protein (structural, NP, P, M, F, HN, and L; non-structural, V and W). After 24 h, cells were infected with La Sota-GFP (MOI = 1) and analyzed at 24 hpi. NC, uninfected cells without transfection, subjected to the same experimental procedures, were included as a negative control. Western blot analysis was performed to assess GFP and viral protein expression. GFP protein levels relative to β-tubulin were quantified by densitometry. Data were normalized to La-GFP, which was set to 1. (**E**) Representative micrographs show fluorescence signal. Scale bar, 500 µm. (**F**) Flow cytometry analysis of BFP and GFP expression. The co-infection rates were calculated as the percentage of cells co-expressing GFP and BFP, divided by the total number of BFP-positive cells for each group. No normalization was performed relative to any specific reference group. (**G**) Comparison of NP expression following plasmid transfection and viral infection. BHK-21 cells were transfected with 1 μg of pCMV-NP-BFP or infected with La Sota-GFP (MOI = 5). At 2, 4, 8, 12, and 24 h post-treatment, cells were harvested for analysis. Cells transfected with 1 μg of pCMV-BFP served as the vector control. Western blot analysis was performed to assess NP protein expression. Data are given as means ± standard deviation from three independent experiments. *P* values were calculated using Student’s *t*-test. An asterisk indicates a comparison with the indicated control. *P < 0.05; **P < 0.01; ***P < 0.001; and ns, not significant.

Previously, we confirmed that NDV SIE initiated as early as 2 hpi of the primary virus. However, WB analysis of La Sota-Cherry-infected cells detected slight HN expression from 2 to 6 hpi, in contrast to the robust signal observed at 8 hpi (Fig. 8A). Consistently, NA activity measurements showed no significant fluctuations between 2 and 6 hpi (Fig. 8B), but compared to 2 hpi of the first NDV, SIE was significantly more pronounced at 6 hpi (Fig. 1). In addition, NDV SIE occurred at 2 hpi of the first virus, but HN was also undetectable by IFA regardless of permeabilization treatment, indicating that HN was not detectably present in either the cytoplasm or at the cell surface at this early stage (Fig. 8C). These findings indicate that factors other than HN contribute to the initiation of NDV SIE. To identify additional viral determinants involved in this process, we generated plasmids expressing the NDV structural proteins (NP, P, M, F, and L) and nonstructural proteins (V and W) using the same strategy as that used for HN. BHK-21 cells were transfected with each construct and challenged with La Sota-GFP 24 h later. WB analysis showed that, in addition to HN, NP markedly reduced GFP accumulation after La Sota-GFP challenge, whereas the other viral proteins had little or no inhibitory effect (Fig. 8D). Consistently, fluorescence imaging revealed a substantial reduction in GFP-positive cells in NP- and HN-expressing cells (Fig. 8E). Flow cytometric analysis further confirmed that NP and HN expression significantly reduced NDV infection rates compared with the other viral proteins tested (Fig. 8F). Furthermore, NP protein was detected by IFA at 2 hpi (Fig. 8C). These data identify NP as an additional NDV factor capable of strongly suppressing secondary NDV infection. To evaluate whether the observed NP-mediated resistance to NDV infection is induced by NP overexpression, we compared NP abundance in plasmid-transfected cells with that in NDV-infected cells. The quantity of NP was significantly lower in the transfection group than that in NDV-infected cells (Fig. 8G). These findings indicate that NP protein expressed during NDV infection is sufficient to mediate SIE. Moreover, when both NP and HN are co-transfected, the exclusion of NDV infection is even more pronounced compared to the individual transfection of either NP or HN (Fig. S6).

**Fig 9:**
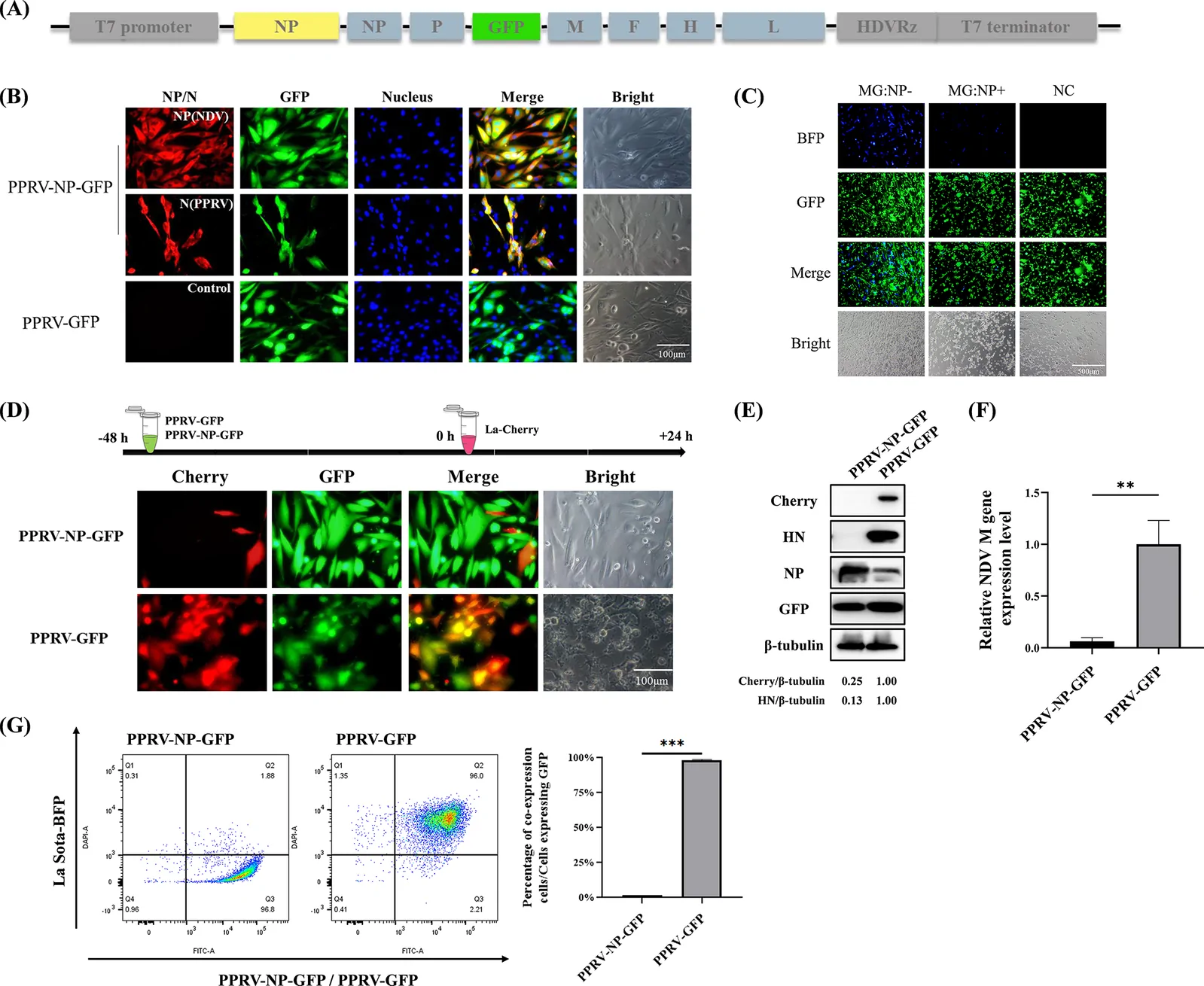
NP expressed from recombinant PPRV-NP-GFP blocks NDV superinfection at the viral level. (**A**) Schematic of recombinant PPRV harboring NDV NP and GFP. (**B**) BHK-21 cells were infected with PPRV-GFP and PPRV-NP-GFP (MOI = 1), respectively. Cells were fixed at 24 hpi. Expression of PPRV N and NDV NP was detected. Cell nuclei were identified with Hoechst 33342. Scale bar, 100 µm. (**C**) BHK-21 cells were infected with PPRV-NP-GFP (MOI = 5). At 24 hpi, the cells were transfected with either pT7-MG-BFP, pCAGGS-P, pCAGGS-L, and pCAGGS-T7 or pT7-MG-BFP, pCAGGS-NP, pCAGGS-P, pCAGGS-L, and pCAGGS-T7. BFP reporter expression was monitored 24–48 h later. NC, cells infected with virus under the same conditions but without plasmid transfection. Representative micrographs show fluorescence signal. Scale bar, 500 µm. (**D**) BHK-21 cells were infected with PPRV-GFP and PPRV-NP-GFP (MOI = 5), respectively, and challenged 48 h later with La Sota-Cherry (MOI = 1); imaging was performed 24 h after the second infection. Representative micrographs show fluorescence signal. Scale bar, 100 µm. (**E**) Western blot analysis was performed on BHK-21 cells 24 h after the second infection to assess viral protein expression. Cherry and HN protein levels relative to β-tubulin were quantified by densitometry. Data were normalized to PPRV-GFP, which was set to 1. (**F**) The relative RNA level of the NDV M gene was determined by qRT-PCR, normalized to the PPRV-GFP group. As an internal control, 28s was used (*n* = 3). (**G**) BHK-21 cells were infected with PPRV-GFP and PPRV-NP-GFP (MOI = 5), respectively, and challenged 48 h later with La Sota-BFP (MOI = 1). Flow cytometry analysis of BFP and GFP expression. The co-infection rates were calculated as the percentage of cells co-expressing GFP and BFP, divided by the total number of GFP-positive cells for each group. Data are given as means ± standard deviation from three independent experiments. *P* values were calculated using Student’s *t*-test. An asterisk indicates a comparison with the indicated control. *P < 0.05; **P < 0.01; ***P < 0.001; and ns, not significant.

To validate NP-mediated SIE at the viral level, we inserted the NDV NP gene into the PPRV-GFP backbone, successfully rescuing recombinant PPRV-NP-GFP virus (Fig. 9A and B). Given the essential role of the NP protein in viral transcription and replication, functionality was assessed using an NDV minigenome system encoding BFP. BFP expression was readily detectable in PPRV-NP-GFP-infected cells even in the absence of the NP expression plasmid during transfection, confirming that the recombinant virus expresses functional NP protein (Fig. 9C). Cells pre-infected with PPRV-NP-GFP or PPRV-GFP were superinfected with NDV La Sota-Cherry or La Sota-BFP. At 24 hpi of NDV, PPRV-NP-GFP-infected cells exhibited dramatically reduced red fluorescence signals compared to PPRV-GFP controls (Fig. 9D). WB analysis showed near-complete ablation of NDV Cherry and HN protein expression in PPRV-NP-GFP pre-infected cells (Fig. 9E). The NDV M gene RNA levels were significantly reduced in PPRV-NP-GFP pre-infected cells compared with PPRV-GFP pre-infected controls (Fig. 9F). Flow cytometry further quantified this exclusion, showing NDV infection rates dropped from 97% in PPRV-GFP pre-infected cells to merely 1.7% (indicating 98.3% exclusion efficiency) in PPRV-NP-GFP pre-infected cells (Fig. 9G). Collectively, these data demonstrated that NP protein also regulates NDV SIE at the viral level.

### NP N-terminal mediates SIE through self-aggregation

**Fig 10:**
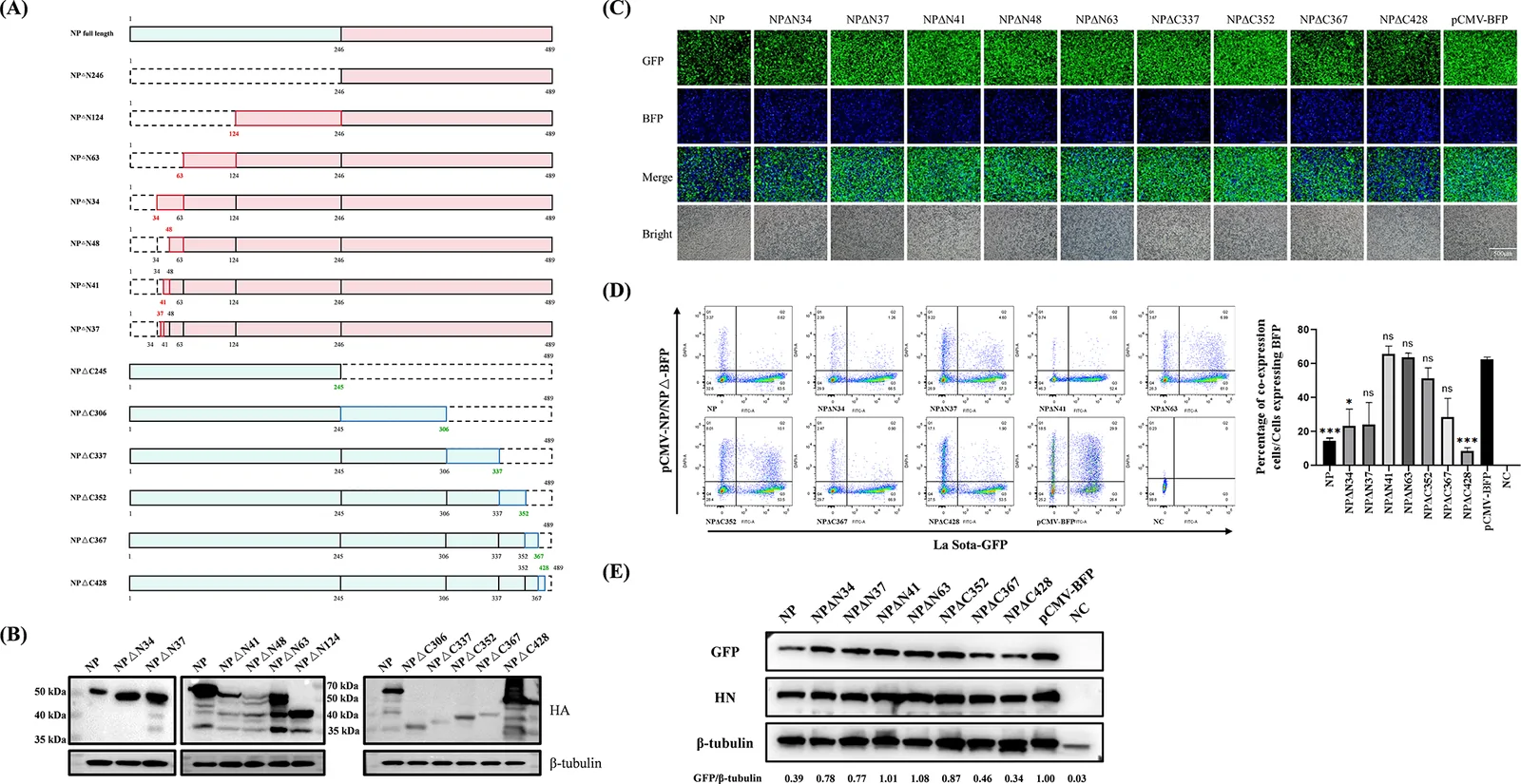
Mapping the SIE-determining regions of NP: residues 1–40 and 353–489 are essential for blocking NDV infection. (**A**) Schematic of NDV NP truncations. (**B**) Western blot analysis was performed to assess the expression of NDV NP and its truncations. (**C**) BHK-21 cells were transfected with 1 µg of plasmids encoding the indicated NP truncations. After 24 h, cells were infected with La Sota-GFP (MOI = 1) and analyzed at 24 hpi. Representative micrographs show fluorescence signal. Scale bar, 500 µm. (**D**) Flow cytometry analysis of BFP and GFP expression. The co-infection rates were calculated as the percentage of cells co-expressing GFP and BFP, divided by the total number of BFP-positive cells for each group. NC, cells treated under the same conditions but without plasmid transfection or viral infection. (**E**) Western blot analysis was performed to assess viral protein expression. GFP protein levels relative to β-tubulin were quantified by densitometry. Data were normalized to pCMV-BFP, which was set to 1. Data are given as means ± standard deviation from three independent experiments. *P* values were calculated using Student’s *t*-test. An asterisk indicates a comparison with the indicated control. *P < 0.05; **P < 0.01; ***P < 0.001; and ns, not significant.

To delineate the functional domains governing NP-mediated NDV SIE, we generated a panel of N-terminal (ΔN34, ΔN37, ΔN41, ΔN48, ΔN63, ΔN124, and ΔN246) and C-terminal truncations (ΔC245, ΔC306, ΔC337, ΔC352, ΔC367, and ΔC428) (Fig. 10A). Western blot confirmed the expression of all constructs except NPΔN246 and NPΔC245 in BHK-21 cells, with molecular weights matching predictions (Fig. 10B). Following transfection and La Sota-GFP challenge, integrated analysis by fluorescence microscopy, immunoblotting, and flow cytometry showed that NPΔN34 retained the ability to slightly suppress NDV infection, whereas NPΔN41 was completely impaired. Similarly, NPΔC352 abolished this function (Fig. 10C through E). Together, these data suggest that the N-terminal region encompassing aa 1–40 is important for NP-mediated SIE, whereas the relevant C-terminal determinant is likely located within a narrower region downstream of aa 352, possibly within aa 353–428.

**Fig 11:**
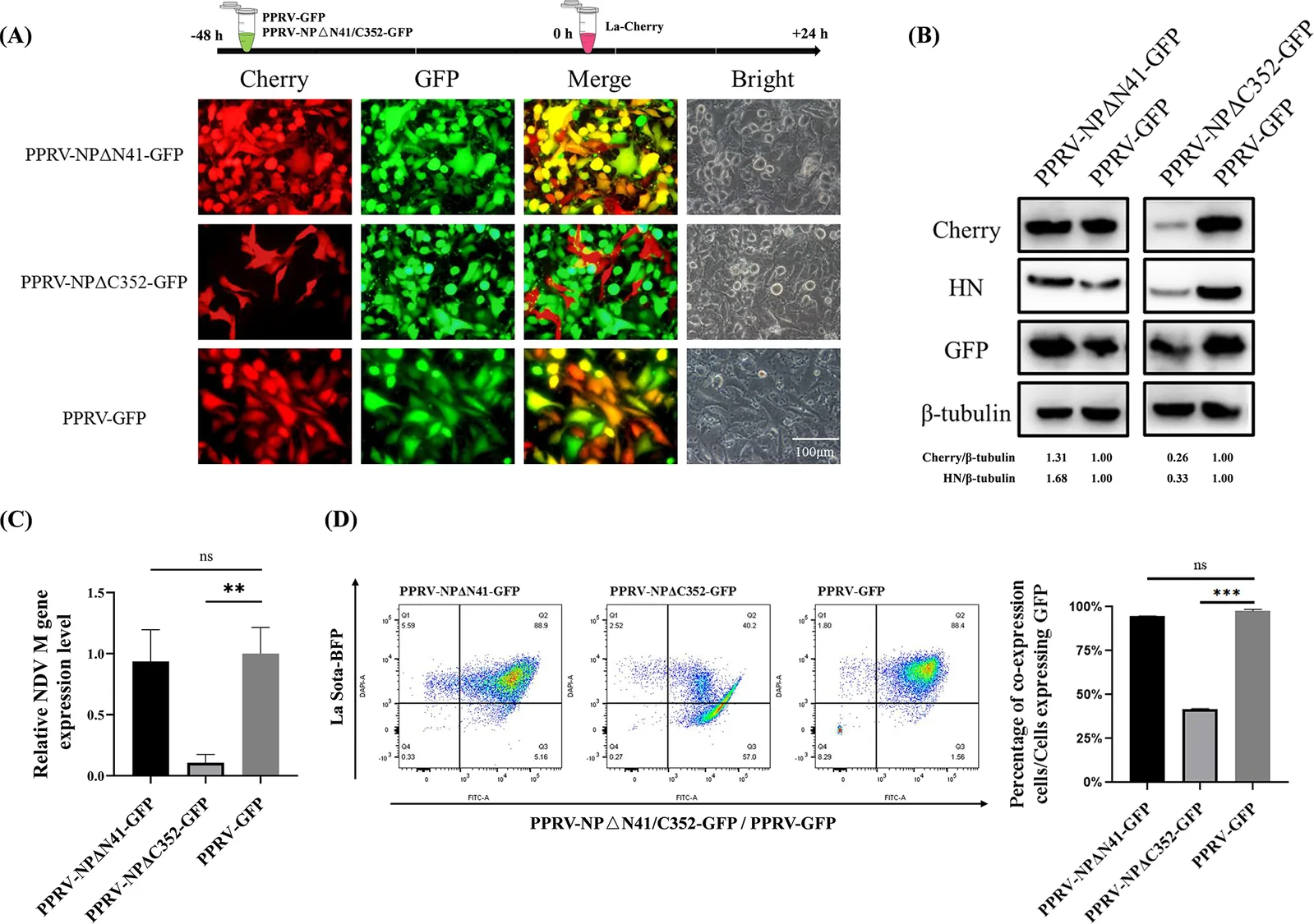
N-terminal 1–40 aa is indispensable for NP-driven SIE. (**A**) BHK-21 cells were infected with PPRV-GFP and PPRV-GFP expressing NDV NP truncations (NPΔN41 and NPΔC352) (MOI = 5), respectively, with PPRV-NP-GFP as a control, and challenged 48 h later with La Sota-Cherry (MOI = 1); imaging was performed 24 h after the second infection. Representative micrographs show fluorescence signal. Scale bar, 100 µm. (**B**) Western blot analysis was performed on BHK-21 cells 24 h after the second infection to assess viral protein expression. Cherry and HN protein levels relative to β-tubulin were quantified by densitometry. Data were normalized to PPRV-GFP, which was set to 1. (**C**) The relative RNA level of the NDV M gene was determined by qRT-PCR, normalized to the PPRV-GFP group. As an internal control, 28s was used (*n* = 3). (**D**) BHK-21 cells were infected with PPRV-GFP and PPRV-GFP expressing NDV NP truncations (NPΔN41 and NPΔC352) (MOI = 5), respectively, with PPRV-NP-GFP as a control, and challenged 48 h later with La Sota-BFP (MOI = 1). The co-infection rates were calculated as the percentage of cells co-expressing GFP and BFP, divided by the total number of GFP-positive cells for each group. No normalization was performed relative to any specific reference group. Data are given as means ± standard deviation from three independent experiments. *P* values were calculated using Student’s *t*-test. An asterisk indicates a comparison with the indicated control. *P < 0.05; **P < 0.01; ***P < 0.001; and ns, not significant.

For viral validation, we engineered recombinant PPRV expressing NPΔN41 or NPΔC352 (PPRV-NPΔN41-GFP and PPRV-NPΔC352-GFP). Cells pre-infected with PPRV-NPΔN41-GFP, PPRV-NPΔC352-GFP, or PPRV-GFP were superinfected with NDV La Sota-Cherry or La Sota-BFP. Compared with PPRV-GFP, PPRV-NPΔN41-GFP-infected cells exhibited abundant Cherry signal, whereas PPRV-NPΔC352-GFP-infected cells showed a markedly reduced number of red fluorescent cells (Fig. 11A). Concordantly, immunoblot analysis of NDV Cherry and HN proteins and RT-qPCR quantification of NDV M gene showed sustained high-level viral protein/genome in PPRV-NPΔN41-GFP-infected cells but significant reduction in PPRV-NPΔC352-GFP groups (Fig. 11B and C). Flow cytometric quantification confirmed 88.9% NDV infection in PPRV-NPΔN41-GFP cells (comparable to controls) versus 40% in PPRV-NPΔC352-GFP cells (Fig. 11D). These results indicate that deletion of the N-terminal 41 aa largely disrupts NP-mediated SIE, whereas deletion from aa 352 onward retains partial inhibitory activity.

**Fig 12:**
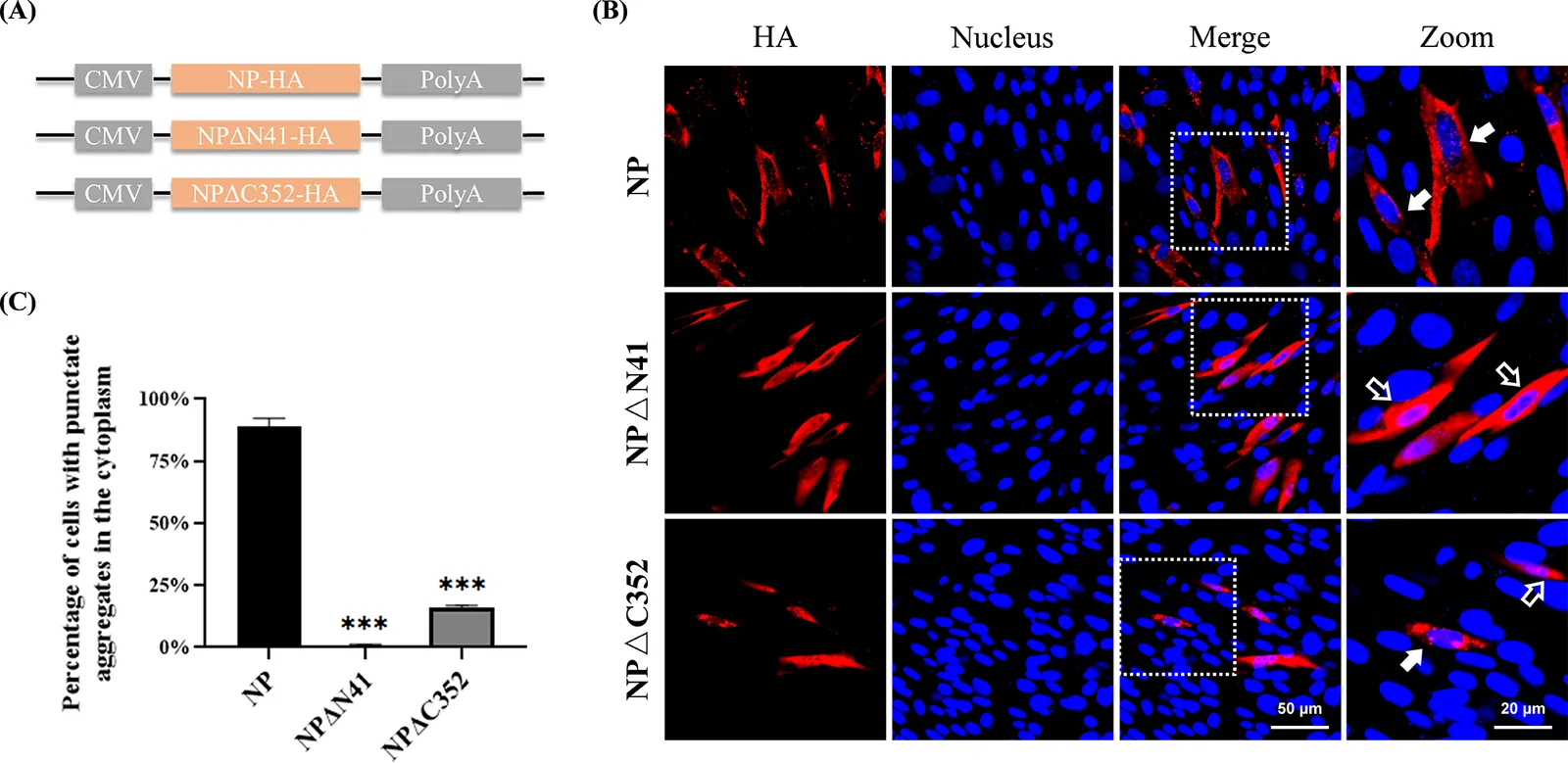
Aggregation propensity dictates NP-mediated SIE. (**A**) Schematic of NDV NP truncation plasmids. (**B**) BHK-21 cells were transfected with 1 µg of pCAGGS-NP-HA, pCAGGS-NPΔN41-HA, and pCAGGS-NPΔC352-HA, respectively. Cells were fixed at 24 h. Expression of NDV NP was detected. Cell nuclei were identified with Hoechst 33342. White and open arrows indicate cells with or without punctate NP, respectively. Scale bar, 50 or 20 µm. (**C**) Quantification of NP aggregation morphology in transfected cells. Data are given as means ± standard deviation from three independent experiments. *P* values were calculated using Student’s *t*-test. An asterisk indicates a comparison with the indicated control. *P < 0.05; **P < 0.01; ***P < 0.001; and ns, not significant.

Given that the NP protein in NDV-infected cells can form aggregates, it is speculated that they may hijack the NP protein of newly invading viruses. To explore the association between aggregation ability and SIE, we constructed HA-labeled eukaryotic expression plasmids NP, NPΔN41, and NPΔC352 (Fig. 12A). These constructs were transfected into BHK-21 cells. Immunofluorescence analysis revealed abundant cytoplasmic puncta in 89% of NP-expressing cells, whereas NPΔC352 showed attenuated aggregation (16%), and NPΔN41 lacked detectable puncta (0.5%), which paralleled their SIE activities (Fig. 12B and C). These results suggested that, in addition to the HN protein through neuraminidase activity, the NP protein can also regulate NDV SIE through aggregation.

## DISCUSSION

In this study, we systematically deciphered the molecular mechanism of NDV SIE and unveiled a previously unrecognized dual-barrier defense system coordinated by the viral surface glycoprotein (HN) and nucleocapsid protein. We demonstrated that NDV establishes SIE within 2 hpi, exhibiting strict intraspecies restriction with no cross-species effect against heterologous viruses such as vesicular stomatitis virus (Fig. 3). This rapid kinetics parallels early exclusion in alphaherpesviruses, and its species specificity confirms dependence on viral-encoded determinants rather than indirect host responses (18).

The core discovery centers on the NA activity of the HN protein as the pivotal effector of SIE. Through construction of chimeric viruses (PPRV-HN-GFP) and NA-functional mutants (HN93, HN160, and HN198), we demonstrate a positive correlation between NA activity levels and SIE efficiency: the NA-deficient mutant (HN198) severely impairs exclusion, whereas the mutant with enhanced NA activity (HN160) exhibits significantly greater exclusion efficacy (Fig. 6 and 7). Mechanistically, during progeny virion budding, the NA activity of the HN protein cleaves sialic acid receptors on the host cell membrane, facilitating viral particle release (43). The enzymatically hydrolyzed sialic acids lose receptor-binding capacity, thereby preventing adsorption of secondary virions and ultimately mediating SIE. The NA of influenza A virus likewise blocks superinfection of infected cells, underscoring the critical dependence of SIE on NA activity (44). Given that both influenza A and NDV recognize SA receptors, Morrison and McGinnes (42) demonstrated that HN expression in cells confers resistance against infection by both NDV and influenza virus. This also accounts for the intraspecies specificity of NDV SIE: heterologous VSV, which enters cells independently of sialic acid receptors, remains unaffected by NDV pre-infection, as confirmed in Fig. 3. Parallel to NDV HN, the VSV G protein functions as a primary SIE effector by competitively occupying host entry pathways, thereby blocking receptor-mediated endocytosis of secondary virions (13). This shared reliance on surface glycoproteins highlights convergent evolution of entry-level exclusion strategies. Likewise, many bacteriophage studies demonstrate receptor blockade as the predominant SIE strategy (45–50).

Notably, SIE was already evident at 2 hpi and became more pronounced over time, but HN remained undetectable at this early stage (Fig. 8A and C). In contrast, NP was readily detected by immunofluorescence at this time point. This result is consistent with the transcriptional attenuation pattern of NDV and suggests that HN expression remains at a low level during early infection. Together, these observations indicate that viral factors other than HN also contribute to NDV SIE. Systematic screening of NDV structural and nonstructural proteins revealed that the NP protein possesses potent NDV exclusion capability comparable to HN (Fig. 8D through F). The chimeric virus PPRV-NP-GFP reduces secondary NDV infection rates to 1.7%, achieving 98.3% exclusion efficiency (Fig. 9D through G). Domain mapping identified the N-terminal (residues 1–40) and C-terminal (residues 353–428) regions as functional cores: truncations to ΔN41 or ΔC352 markedly diminish NP self-aggregation capacity (Fig. 12B and C), paralleled by attenuated SIE (Fig. 10 and 11). Critically, the complete aggregation defect in ΔN41 (0.5% aggregation-positive cells) directly correlates with abolished SIE function (Fig. 11 and 12). Although the inhibitory magnitude of NPΔN41 differed between the plasmid-based and recombinant PPRV-based systems, likely reflecting differences in expression efficiency and uniformity, both approaches consistently showed that deletion of the N-terminal 41 aa markedly impairs NP-mediated SIE. Future studies using finer C-terminal deletions and double-truncation mutants lacking both the N- and C-terminal determinants will be important to determine whether these regions act independently or cooperatively in mediating NP aggregation and SIE.

This indicates that NP, as the core nucleocapsid component, hijacks NP subunits of newly invading viruses via cytoplasmic aggregate formation, disrupting viral replication complex assembly and subsequent genome transcription/replication. Significantly, since NP is abundantly expressed immediately post-infection, it could constitute the primary defense barrier prior to substantial HN accumulation. This hijacking strategy exploits a conserved molecular mechanism. Transcriptional attenuation of the viral genome causes the NP to accumulate at substantially higher levels than other viral proteins. This results in an excess of unassembled NP that fails to incorporate into functional replication complexes with the P, L, and viral RNA. Due to their intrinsic self-aggregation capacity, these surplus NP proteins autonomously form cytoplasmic puncta or aggregates. This phenomenon is conserved across Paramyxoviridae: as demonstrated in heterologous systems lacking viral RNA or auxiliary viral components, the N/NP proteins of Sendai virus, measles virus, and NDV self-assemble into flexible helical or hairpin-like structures (31, 51–53). Crucially, these aggregates lack functional competence in viral replication and transcription.

In turnip crinkle virus, the p28 protein mediates SIE by forming intracellular aggregates or inclusion bodies. These aggregates efficiently sequester p28 proteins derived from secondary virions, thereby preventing their incorporation into functional viral replication complexes (19). Concurrently, as an essential component of viral replication, p28 collaborates with host factors and the viral p88 protein to assemble functional viral replication complexes (54–56). Remarkably, NDV NP protein employs a strikingly similar strategy: it hijacks NP subunits from secondary viruses via self-aggregation, thereby inhibiting the assembly of viral replication complexes and ultimately mediating SIE. Similarly, this strategy is employed by the SIE factors of Tobacco mosaic virus (TMV) and Soybean mosaic virus (SYNV). During primary infection, these viruses abundantly produce their respective viral assembly factors—TMV coat protein and SYNV M protein. These factors function to coat or sequester the genomic RNAs of secondary-invading TMV and the genomic ribonucleoprotein complexes of secondary-invading SYNV, thereby preventing superinfection (17, 20).

**Fig 13:**
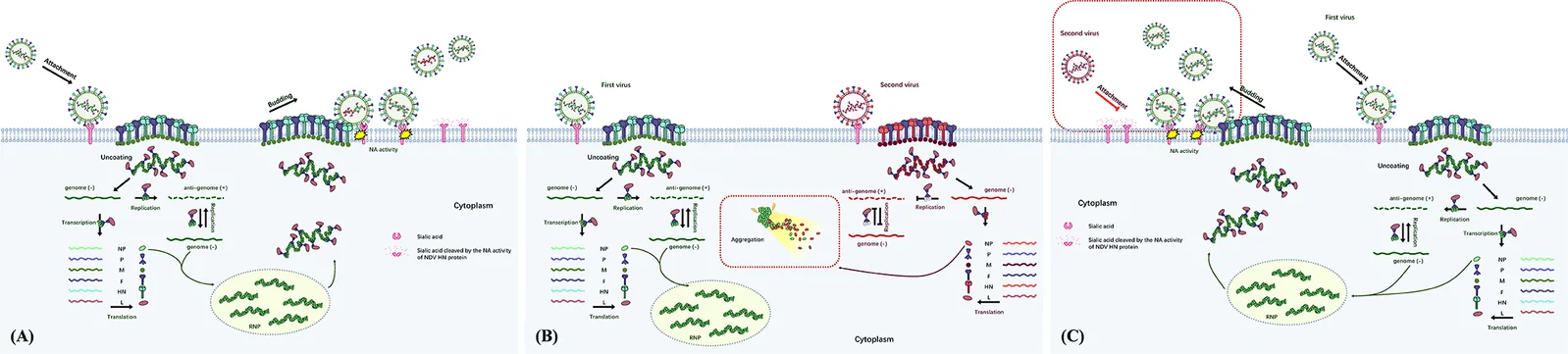
A model of dual-barrier mechanism-mediated NDV SIE. (**A**) Primary infection begins when the HN glycoprotein binds sialic acid receptors and triggers membrane fusion, releasing the ribonucleoprotein into the cytoplasm. Early transcription and translation rapidly elevate NP levels; once a threshold is reached, genome replication starts, and nascent RNPs are assembled with NP, P, and L. These complexes traffic to the plasma membrane where budding occurs, aided by HN neuraminidase activity that cleaves sialic acid bonds and liberates progeny virions. (**B**) During the early phase, surplus NP molecules oligomerize via their N-terminal domain and form cytoplasmic sequestration complexes that capture incoming NP subunits from secondary virions, thereby aborting replication complex assembly of any subsequently invading virus. (**C**) Later, HN accumulated at the cell surface provides a second, membrane-level barrier: continuous NA activity erases sialic acid receptors and physically prevents adsorption of later virions. This two-step, spatiotemporally partitioned mechanism secures uninterrupted superinfection exclusion throughout the NDV replication cycle.

Collectively, NDV deploys two spatially and temporally distinct barriers. NP forms an early intracytoplasmic barrier by disrupting viral replication machinery through aggregate-mediated hijacking, while HN establishes a late surface barrier by blocking secondary virion entry via receptor destruction (Fig. 13). This cooperative system ensures robust, staged SIE throughout the infection cycle. Conventional understanding holds that SIE in paramyxoviruses is predominantly mediated by receptor destruction. This study pioneers the identification of a novel SIE mechanism driven by NP aggregation, fundamentally expanding the conceptual scope of viral self-limitation strategies. The dual-pronged strategy prevents resource competition from co-invading viral variants, particularly for finite cellular assets, such as nucleotide pools, ribosomes, and replication compartments, while enforcing physical segregation between primary and secondary viral genomes. By minimizing opportunities for genetic recombination or reassortment, SIE safeguards the integrity and transmission fidelity of the founding viral population.

## MATERIALS AND METHODS

### Cells and viruses

The chicken embryo fibroblast cell line (DF-1) and baby hamster kidney cell line (BHK-21) were obtained from the American Type Culture Collection (ATCC, Manassas, VA, USA). Both cell lines were cultured in Dulbecco’s minimal essential medium (DMEM; Gibco, C11995500BT) supplemented with 10% fetal bovine serum (FBS; ZETA, France) and grown in a 37°C incubator supplied with 5% CO_2_.

NDV La Sota-mCherry, La Sota-GFP, La Sota-BFP, and C22-GFP were stored in our laboratory and propagated in the allantoic cavity of 9- to 11-day-old SPF embryonated chicken eggs. VSV-GFP was stored in our laboratory and propagated in BHK-21 cells. Virus titers (50% tissue culture infectious doses/mL [TCID_50_/mL]) were calculated using the Reed and Muench method. In prior work, we established a reverse genetics system for the PPRV vaccine strain (Nigeria75/1). By inserting the GFP gene between the P and M genes, we rescued the rPPRV-GFP (GFP-expressing) strain, which is stored at −80°C.

### Cloning and mutagenesis

The HN of NDV strain F48E9 was amplified from viral cDNA and inserted between the XhoI and NotI sites of the intermediate vector pCI-neo to generate pCI-HN. A fragment spanning the CMV promoter to the SV40 poly(A) signal was then PCR-amplified from pCI-HN and cloned into BssHII/PacI-digested, laboratory-modified pCMV-BFP, yielding pCMV-HN-BFP. In this construct, HN and BFP are driven by independent CMV promoters and terminated by separate poly(A) signals, ensuring autonomous expression. Point mutations were introduced into pCMV-HN-BFP by overlap-extension PCR; the HN gene was split into two fragments, each amplified with mutagenic primers, fused, and cloned into the XhoI/NotI sites of pCMV-BFP to generate the desired mutant plasmids. Expression plasmids for NP (full-length and truncations), P, M, F, HN, L, V, and W were constructed using the same cloning strategy.

Starting from the laboratory-maintained PPRV-GFP reverse-genetics backbone, we amplified the F48HN from F48E9 cDNA and inserted it between the leader sequence and the N gene of the PPRV genome. Owing to a paucity of unique restriction sites, the leader-5′ UTR, F48HN, and the N-gene 3′ UTR were first fused by overlap-extension PCR. The resulting fragment was cloned into BssHII/SpeI-digested pCMV-PPRV-TRANS to generate pCMV-HN-PPRV-TRANS. This cassette was excised with BssHII/NotI and ligated into the full-length vector pT7-PPRV-GFP, yielding pT7-PPRV-HN-GFP, which was verified by sequencing. Point-mutant HN was introduced identically, producing pT7-PPRV-HN93-GFP, pT7-PPRV-HN160-GFP, and pT7-PPRV-HN198-GFP. Full-length PPRV plasmids encoding NDV NP or its truncation mutants were constructed using the same strategy.

### Rescuing viruses

BHK-21 cells were seeded in 6-well plates and grown to full confluence before transfection. Each well received a mixture of 0.6 µg pCAGGS-T7, 1.2 µg pCMV-NPL, and 1.2 µg full-length pT7-PPRV-HN-GFP prepared as follows: plasmids were diluted in 100 µL Opti-MEM (Gibco, 31985070), mixed gently, combined with 6 µL TurboFect transfection reagent (Thermo Scientific, R0531), incubated at room temperature (RT) for 15 min, and then added dropwise to cells that had been rinsed twice with Opti-MEM and overlaid with 2 mL 10% FBS MEM. Cultures were maintained at 37°C in 5% CO_2_; green fluorescence was monitored every 24 h by inverted microscopy. When GFP-positive cells were abundant and slight detachment was evident (typically 48–72 h post-transfection), the cells were passaged to amplify the virus. For passage, fresh 6-well plates containing 2 mL of 10% FBS MEM were prepared; rescue wells were rinsed with 500 µL trypsin, incubated with 400 µL trypsin for 2 min, and neutralized with 400 µL 10% FBS MEM. Cells were triturated to a single-cell suspension, distributed 1:3 into new wells, and observed every 12 h. After three to four passages, when >80% of cells expressed high levels of GFP, plates were subjected to three freeze-thaw cycles at −80°C to release intracellular virions. Lysates were clarified by brief centrifugation and stored at −80 °C until use.

### Infections

Virus was thawed on ice and titrated by TCID_50_ to permit accurate MOI calculations. After removal of growth medium and a single PBS rinse, cells were inoculated with virus diluted in DMEM to the predetermined titer. Plates were incubated at 37°C in 5% CO_2_ for 1 h with gentle rocking every 15 min. The inoculum was then aspirated and replaced with 2% FBS DMEM; cultures were returned to the incubator for the remainder of the infection period.

### RNA isolation and real-time PCR analysis

Total RNA was extracted from cells using RNAiso Plus reagent (Takara, 9109). RNA was then reverse transcribed using PrimeScript RT Master Mix (Takara, RR036A). Real-time quantitative PCR assays were carried out using an ABI 7500 System (Applied Biosystems, Warrington, UK) and 2× Fast qPCR Master Mix (DiNing, DN2055). The primer sequences used in this study are as follows: GFP, forward, 5′-ATGGCCGACAAGCAGAAGAA; GFP, reverse, 5′-CTCAGGTAGTGGTTGTCGGG; Cherry, forward, 5′-TTCATGTACGGCTCCAAGGC; Cherry, reverse, 5′-CTGCTTGATCTCGCCCTTCA; 28s, forward, 5′-GGTATGGGCCCGACGCT; 28s, reverse, 5′-CCGATGCCGACGCTCAT; NDV M, forward, 5′-AAGAGGTGCACGGACTAAGC; NDV M, reverse, 5′-CGTTGGGTACCTGCTTGGAT.

### Flow cytometry

Flow cytometry analysis was performed to quantify the cells expressing GFP and BFP. Culture medium was aspirated, and monolayers were washed twice with PBS. Adherent cells were detached by incubation with 0.25% trypsin (200 µL per well of a 24-well plate) for an optimized period, after which an equal volume of DMEM containing 10% FBS was added to terminate digestion. Cells were collected into 1.5 mL tubes, pelleted at 1,500 rpm for 3 min at RT, and washed once with 500 µL PBS under the same centrifugation conditions. The resulting pellet was resuspended in an appropriate volume of PBS and filtered through a 300-mesh sieve into flow cytometry tubes. Acquisition was performed on the BD FACS Aria III High Speed Cell Sorter (BD Biosciences, San Diego, CA, USA) using FITC and DAPI channels for GFP and BFP detection, respectively. Each sample was prepared in triplicate and analyzed with FlowJo v10.6.

### Syncytium formation

To assess the fusion activity of HN mutants, BHK-21 cells were seeded in 24-well plates and grown to confluence (12–16 h). HN-mutant plasmids (0.5 µg) were co-transfected with pCAGGS-F (0.5 µg) using 2 µL TurboFect in 100 µL Opti-MEM. Syncytium formation was evaluated qualitatively (presence/absence) at 24 h post-transfection under an inverted microscope. Syncytia were defined as obvious cell-cell fusion forming large fused cell masses with indistinct cell boundaries.

For virus-encoded HN, BHK-21 cells infected with PPRV-HN-GFP (or PPRV-GFP alone) received 0.5 µg pCAGGS-F, with an additional control co-transfected with pCAGGS-HN (0.5 µg). At 24 h post-transfection, the presence or absence of syncytia was compared across groups to determine whether HN expressed by recombinant PPRV is fusion-competent and whether HN mutations alter fusion activity.

### Hemadsorption (HAd) assay

HAd activity was quantified to verify functional expression of NDV wild-type and mutant HN proteins and was performed according to published methods (57). Cells transfected with HN-expressing plasmids or infected with recombinant PPRV-GFP encoding NDV HN were seeded in 24-well plates and cultured for 12–16 h. After two washes with ice-cold PBS, 500 µL of 2% (vol/vol) chicken red blood cells (CRBCs) in PBS was added to each well and incubated on ice for 1 h to allow adsorption. Subsequently, the monolayers were washed thoroughly to remove unbound CRBCs. The remaining bound CRBCs were lysed with 115 µL Red Blood Cell Lysis Buffer (Solarbio, China, R1010) for 5 min. Cells were detached, pooled into microcentrifuge tubes, and clarified by centrifugation at 12,000 rpm for 5 min at 4°C. A 100 µL aliquot of the supernatant was transferred to a 96-well plate, and absorbance at 415 nm was measured.

### Neuraminidase assay

NA activity was measured in BHK-21 cells expressing wild-type or mutant HN proteins. Cells were transfected with HN-encoding plasmids or infected with recombinant PPRV-GFP viruses carrying NDV HN or its derivatives, prepared as described above. After transfection or infection, monolayers were detached with 50 mM EDTA in PBS, pelleted at 3,000 × *g* for 5 min at 4°C, and washed once with ice-cold PBS to remove EDTA. Cell pellets were lysed in 20 µL RIPA buffer (Solarbio, China) by three freeze-thaw cycles at −80°C followed by centrifugation at 12,000 rpm for 5 min at 4°C. Then, the remaining steps were performed according to the manufacturer’s instructions for the neuraminidase assay kit (Beyotime, P0306, China). Samples collected from virus-free cells served as controls. The samples were transferred into a black-walled, clear-bottom 96-well plate. The fluorescence of the cleaved substrate was measured at excitation and emission wavelengths of 356 and 450 nm, respectively, using a microplate spectrophotometer (Infinite 200 PRO Nano Quant, Tecan, Switzerland).

### Minigenome assay

BHK-21 cells were infected with PPRV-NP-GFP (MOI = 5) in 24-well plates. At 24 hpi, the cells were transfected with either 0.5 µg pT7-MG-BFP, 0.2 µg pCAGGS-P, 0.1 µg pCAGGS-L, and 0.5 µg pCAGGS-T7 or 0.5 µg pT7-MG-BFP, 0.2 µg pCAGGS-NP, 0.2 µg pCAGGS-P, 0.1 µg pCAGGS-L, and 0.5 µg pCAGGS-T7 (as a positive control). BFP reporter expression was monitored 24–48 h later.

### Western blot analysis

The harvested cells were lysed in RIPA lysis buffer containing protease inhibitors. Then, 5× SDS loading buffer was added to the lysates before boiling for 10 min, then separated by SDS-PAGE on 10% polyacrylamide gels, and transferred onto 0.22 μm polyvinylidene difluoride membranes (Millipore, USA). Membranes were blocked with 10% skim milk for 2 h at RT before incubation with appropriate primary antibodies overnight at 4°C: monoclonal antibody against NDV-NP (provided by Chan Ding, Shanghai Veterinary Research Institute), NDV-HN monoclonal antibody (2G8) produced in our lab in a previous study (58), PPRV N protein monoclonal antibody (provided by Xuefeng Qi, Northwest A&F University), HA-Tag (Cell Signaling Technology, #3724), GFP tag monoclonal antibody (Proteintech, 66002-1-Ig), Cherry polyclonal antibody (Proteintech, 26765-1-AP), β-tubulin (Proteintech, 66240-1-Ig), and then, the membranes were washed five times with TBST (TBS buffer containing 0.05% Tween-20) for 5 min each time. Subsequently, the membranes were incubated with secondary antibody (HRP-conjugated goat anti-mouse/rabbit IgG; Diyibio, China) for 1 h. After washing five times with TBST, an ECL super sensitive kit (DiNing, Beijing, China) was used to visualize the membrane using a chemiluminescence imager (MiniChemi610; Sagecreation, Beijing, China).

### Indirect immunofluorescence assay

For indirect immunofluorescence assay, cells were washed three times with PBS, fixed in 4% paraformaldehyde (diluted in PBS) for 30 min, and then permeabilized with 0.2% Triton X-100 in PBS for 15 min. Samples were blocked with 1% bovine serum albumin for 1 h at 37°C and incubated with the appropriate primary antibodies at 4°C overnight. Then, the cells were incubated with goat anti-mouse IgG H&L (Alexa Fluor 594, Abcam, ab150116) or goat anti-rabbit IgG H&L (Alexa Fluor 488, Abcam, ab150077) for 1 h at 37°C. Next, the cells were incubated with Hoechst 33342 Stain Solution (Solarbio, C0030; 1:1,000) for 10 min. Between and after each incubation step, the cells were washed three times with PBST. Finally, cells were visualized under a fluorescence microscope (Olympus, Japan).

### Assessment of NP truncation mutant aggregation morphology

Because the existing pCMV-NPΔN41-BFP and pCMV-NPΔC352-BFP vectors emit blue fluorescence that precludes nuclear counterstaining, the full-length NP, NPΔN41, and NPΔC352 were cloned into pCI-neo with a C-terminal HA tag, generating pCI-NP-HA, pCI-NPΔN41-HA, and pCI-NPΔC352-HA. BHK-21 cells seeded in 24-well plates were transfected with 1 µg of each plasmid. At 24 h post-transfection, cells were fixed and processed for HA-specific immunofluorescence. Ten random fields per construct were imaged, and the percentage of cells displaying NP aggregates was calculated relative to the total number of HA-positive cells.

### Statistical analysis

Statistical analysis was performed using GraphPad Prism 9 software (GraphPad Software, Inc., La Jolla, CA, USA). All results were presented as the mean ± standard deviation. The two-tailed Student’s unpaired *t*-test was used to determine statistical significance, as indicated by asterisks in the figures: ns, not significant; *P < 0.05; **P < 0.01; and ***P < 0.001.

## Data Availability

All study data are included in the main text and supplemental material.

## SUPPLEMENTAL MATERIAL

Fig. S1 mbio.03545-25-s0001.tif La Sota-GFP excludes La Sota-Cherry infection in BHK-21 cells.

Fig. S2 mbio.03545-25-s0002.tif La Sota-GFP excludes La Sota-Cherry infection in DF-1 cells.

Fig. S3 mbio.03545-25-s0003.tif SIE also occurs between attenuated and virulent NDV strains.

Fig. S4 mbio.03545-25-s0004.tif Rescue and characterization of PPRV-GFP expressing the NDV HN protein.

Fig. S5 mbio.03545-25-s0005.tif Rescue and characterization of PPRV-GFP expressing the mutant NDV HN protein.

Fig. S6 mbio.03545-25-s0006.tif Synergistic effect of co-transfection of NP and HN on NDV SIE.

Legends mbio.03545-25-s0007.pdf Supplemental figure legends.

ASM does not own the copyrights to Supplemental Material that may be linked to, or accessed through, an article. The authors have granted ASM a non-exclusive, world-wide license to publish the Supplemental Material files. Please contact the corresponding author directly for reuse.
