# Supplementary material for: Dual-barrier mechanism of superinfection exclusion in Newcastle disease virus: nucleoprotein and hemagglutinin-neuraminidase cooperate to block secondary infection
Source: mBio. 2026 Oct 14;17(11):e03545-25. doi: 10.1128/mbio.03545-25 (PMC13753825; doi:10.1128/mbio.03545-25)
Supplement: Supplementary file 1 [file mbio.03545-25-s0007.pdf]

FIG S1 La Sota-GFP excludes La Sota-Cherry infection in BHK-21 cells. (A) Schematic representation of the virus infection. BHK21 cells were infected with La Sota-GFP (MOI=5) and challenged with La Sota-Cherry (MOI=1) at 0, 2, 4, 6 or 8 h intervals; imaging and sampling were performed 24 h after the second or single virus infection. Positive controls consisted of single infections with La Sota-GFP(MOI=1) or La Sota-Cherry (MOI=5). NC, cells treated with the same conditions but not infected with the virus. **(B)** Representative micrographs showing fluorescence signal. The cells were fixed and nuclei were stained with Hoechst 33342. Scale bar, 500 μm. **(C)** Percentage of Cherry-positive cells counted from 5 random fields (n=3). **(D)** The RNA of fluorescent reporter gene was detected by qRT-PCR. 28s was used as a loading control (n=3). **(E)** Western blot analysis was performed on BHK-21 cells 24 h after the second infection to assess viral protein expression. Cherry protein levels relative to β-tubulin were quantified by densitometry. Data were normalized to La-Cherry, which was set to 1. Data are given as means ± standard deviation (SD) from three independent experiments. P values were calculated using Student's t-test. An asterisk indicates a comparison with the indicated control. $^*P < 0.05$; $^{**}P < 0.01$; $^{***}P < 0.001$; ns: not significant.

FIG S2 La Sota-GFP excludes La Sota-Cherry infection in DF-1 cells. (A) Schematic representation of the virus infection. DF-1 cells were infected with La Sota-GFP (MOI=5) and challenged with La Sota-Cherry (MOI=1) at 0, 2, 4, 6 or 8 h intervals; imaging and sampling were performed 24 h after the second infection. Controls consisted of single infections with La Sota-GFP (MOI=5) or La Sota-Cherry (MOI=1). NC, cells treated with the same conditions but not infected with the virus. **(B)** Representative micrographs showing fluorescence signal. The cells were fixed and nuclei were stained with Hoechst 33342. Scale bar, 500 μm. **(C)** Percentage of Cherry-positive cells counted from 5 random fields (n=3). **(D)** The RNA of fluorescent reporter gene was detected by qRT-PCR. 28s was used as a loading control (n=3). **(E)** Western blot analysis was performed on DF-1 cells 24 h after the second infection to assess viral protein expression. Cherry protein levels relative to β-tubulin were quantified by densitometry. Data were normalized to La-Cherry, which was set to 1. Data are given

as means ± standard deviation (SD) from three independent experiments. *P* values were calculated using Student's *t*-test. An asterisk indicates a comparison with the indicated control. *$P < 0.05$; **$P < 0.01$; ***$P < 0.001$; ns: not significant.

FIG S3 SIE also occurs between attenuated and virulent NDV strains. (A) Schematic representation of the virus infection. BHK-21 cells were infected with La Sota-Cherry (MOI=5) and challenged with C22-GFP (MOI=1) at 0, 2, 4, 6 or 8 h intervals; imaging and sampling were performed 24 h after the second infection. Controls consisted of single infections with La Sota-Cherry (MOI=5) or C22-GFP (MOI=1). NC, cells treated with the same conditions but not infected with the virus. **(B)** Representative micrographs showing fluorescence signal. The cells were fixed and nuclei were stained with Hoechst 33342. Scale bar, 500 μm. **(C)** Percentage of GFP-positive cells counted from 5 random fields (n=3). **(D)** The relative RNA of fluorescent reporter gene was detected by qRT-PCR. 28s was used as a loading control (n=3). **(E)** Western blot analysis was performed on BHK-21 cells 24 h after the second infection to assess viral protein expression. GFP protein levels relative to β-tubulin were quantified by densitometry. Data were normalized to C22-GFP, which was set to 1. Data are given as means ± standard deviation (SD) from three independent experiments. *P* values were calculated using Student's *t*-test. An asterisk indicates a comparison with the indicated control. *$P < 0.05$; **$P < 0.01$; ***$P < 0.001$; ns: not significant.

FIG S4 Rescue and characterization of PPRV-GFP expressing the NDV HN protein. (A) Schematic of recombinant PPRV harboring F48E9 HN and GFP. **(B)** BHK-21 cells were infected with PPRV-GFP and PPRV-HN-GFP (MOI=1), respectively. Cells were fixed at 24 hpi. Expression of PPRV N and NDV HN was detected. Cell nuclei were identified with Hoechst 33342. Scale bar, 100 μm. **(C)** Western blot analysis was performed to confirm expression of the recombinant PPRV-HN-GFP viral proteins. **(D)** BHK-21 cells were infected with either PPRV-GFP or PPRV-HN-GFP (MOI=1). At 48 hpi, PPRV-GFP-infected cells were transfected with 0.5 μg of F48E9 F-expressing plasmid alone or with 0.5 μg of F48E9 HN-expressing plasmid, whereas PPRV-HN-GFP-infected cells were transfected with 0.5 μg of the F plasmid only. Syncytium formation was assessed 24 h post-transfection. Scale bar, 100 μm. **(E)** HAd

activity of PPRV-HN-GFP was determined (n=3). **(F)** NA activity of PPRV-HN-GFP was determined (n=3). Data are given as means ± standard deviation (SD) from three independent experiments. *P* values were calculated using Student's *t*-test. An asterisk indicates a comparison with the indicated control. *$P < 0.05$; **$P < 0.01$; ***$P < 0.001$; ns: not significant.

FIG S5 Rescue and characterization of PPRV-GFP expressing the mutant NDV HN protein. (A) BHK-21 cells were infected with PPRV-GFP, PPRV-HN93-GFP, PPRV-HN160-GFP or PPRV-HN198-GFP (MOI=1), respectively. Cells were fixed at 24 hpi. Expression of PPRV N and NDV HN was detected. Cell nuclei were identified with Hoechst 33342. Scale bar, 100 μm. **(B)** NA activity of PPRV-GFP expressing NDV HN mutants was determined (n=3). Data are normalized to wild-type HN activity (set at 100%). **(C)** HAd activity of PPRV-GFP expressing NDV HN mutants was determined (n=3). Data are normalized to wild-type HN activity (set at 100%). Data are given as means ± standard deviation (SD) from three independent experiments. *P* values were calculated using Student's *t*-test. An asterisk indicates a comparison with the indicated control. *$P < 0.05$; **$P < 0.01$; ***$P < 0.001$; ns: not significant.

FIG S6 Synergistic Effect of Co-transfection of NP and HN on NDV SIE. BHK-21 cells were co-transfected with 1 μg of pCMV-NP-BFP plus 1 μg of pCMV-BFP; 1 μg of pCMV-HN-BFP plus 1 μg of pCMV-BFP; or 1 μg each of pCMV-NP-BFP and pCMV-HN-BFP. Cells transfected with 2 μg of pCMV-BFP served as the vector control. After 24 h, cells were infected with La Sota-GFP (MOI=1) and analyzed at the 24 hpi. NC, cells subjected to the same procedures but without plasmid transfection and viral infection. **(A)** Representative fluorescence images. Scale bar, 500 μm. **(B)** Western blot analysis of GFP and viral protein expression. GFP protein levels relative to β-tubulin were quantified by densitometry. Data were normalized to cells only with La Sota-GFP infection.
